# Real-world pharmacovigilance of ofatumumab in multiple sclerosis: a comprehensive FAERS data analysis

**DOI:** 10.3389/fphar.2024.1521726

**Published:** 2025-01-23

**Authors:** Hui Chen

**Affiliations:** Department of Neurology, Neurological Diagnosis and Treatment Center, Jinan Third People’s Hospital, Jinan, China

**Keywords:** ofatumumab, multiple sclerosis, pharmacovigilance, FAERS database, adverse events, disproportionality analysis

## Abstract

**Background:**

Ofatumumab, a fully human monoclonal antibody targeting CD20, is approved for the treatment of relapsing multiple sclerosis. Comprehensive real-world safety data are crucial for informing clinical practice.

**Methods:**

The FDA Adverse Event Reporting System database was utilized to perform a disproportionality analysis, covering reports from Q3 2020 to Q2 2024, in which ofatumumab was identified as the primary suspected drug. Statistical approaches used included the Reporting Odds Ratio, Proportional Reporting Ratio, Bayesian Confidence Propagation Neural Network, and Multi-item Gamma Poisson Shrinker. The timing of adverse events was assessed using the Weibull distribution model to highlight temporal risk patterns.

**Results:**

Known adverse reactions, such as injection site reactions and upper respiratory tract infections, displayed positive signals. Additionally, novel off-label adverse events, including brain fog, muscle spasms, and mood alterations, were identified, marking the first real-world evidence of these potential risks. Temporal analysis revealed that most adverse events occurred within the first month of treatment, indicating an early risk phase. Subgroup analysis demonstrated notable differences in adverse event profiles by gender and age, with males more prone to hyperhidrosis and older patients more susceptible to neurological symptoms.

**Conclusion:**

This real-world analysis of ofatumumab provides important safety insights, confirming known adverse reactions and identifying additional potential risks. Early and tailored monitoring protocols during the initial treatment phase, including regular neurological and psychiatric assessments, are recommended to optimize patient safety and outcomes. Prospective studies are recommended to validate these results and explore the underlying mechanisms.

## 1 Introduction

Ofatumumab, a fully human monoclonal antibody targeting CD20, has been approved for treating relapsing forms of multiple sclerosis (MS). By specifically depleting B lymphocytes, it effectively reduces B cell-mediated inflammatory activity, thereby controlling disease progression and lowering relapse rates (McGinley et al., 2021). MS is a chronic inflammatory demyelinating disorder of the central nervous system (CNS), marked by recurrent episodes of neurological impairment that may progress to irreversible neurodegeneration (Thompson et al., 2018). Unlike conventional disease-modifying therapies, the subcutaneous administration of ofatumumab offers improved convenience, potentially enhancing patient adherence. However, data on its long-term safety in broader patient populations remain limited, with a notable lack of comprehensive real-world assessments (Hauser et al., 2020).

Clinical trials have shown promising results regarding the efficacy and safety profile of ofatumumab, the strict inclusion criteria of these trials often create study cohorts that do not fully reflect the diversity of real-world patient populations, limiting the generalizability of their safety findings (Hauser et al., 2020; Katkade et al., 2018; Kira et al., 2022). Real-world data, in contrast, encompass a wider range of patients, including those with comorbidities, different demographic characteristics, and varying treatment adherence. This diversity provides a more comprehensive understanding of a drug’s safety profile across heterogeneous populations, which is particularly critical for identifying rare or unexpected adverse events that may not emerge in controlled clinical settings. Moreover, real-world studies allow for longer observation periods, capturing delayed or cumulative adverse effects that may be missed in shorter clinical trials (Thompson et al., 2018). According to prescribing information, common adverse reactions to ofatumumab include an increased risk of infections, reductions in immunoglobulin levels, injection site reactions, and potential hypersensitivity (McGinley et al., 2021). A detailed assessment of real-world safety data is essential to provide more comprehensive guidance for clinical practice (Blonde et al., 2018).

Temporal patterns of adverse events provide crucial insights into the timing of drug-related risks, enabling clinicians to identify high-risk periods and implement targeted monitoring strategies. For ofatumumab, understanding the temporal clustering of adverse events, especially during the initial treatment phase, is essential for optimizing patient safety. By focusing on the onset timing of adverse reactions, this study aims to uncover clinically relevant patterns that can guide early intervention and personalized risk management.

This study utilized the FDA Adverse Event Reporting System (FAERS) to perform a thorough evaluation of the adverse event profile of ofatumumab in patients with MS. By examining the reporting frequency, types of AEs, and their temporal patterns, the study aimed to detect potential positive safety signals and provide enhanced safety insights to support clinical risk management.

## 2 Materials and methods

### 2.1 Data sources and data cleaning

This study utilized the FAERS database, which consists of seven key files: Demographic Information (DEMO), Drug Information (DRUG), Adverse Reaction Information (REAC), Outcome Information (OUTC), Report Source Information (RPSR), Therapy Information (THER), and Indication Information (INDI). As a voluntary reporting system (SRS), FAERS gathers unsolicited submissions from patients, healthcare professionals, and pharmaceutical manufacturers, offering valuable data for real-world analyses of adverse drug reactions. This study included all adverse event records where ofatumumab was recognized as the primary suspected drug, from Q3 2020, when it received FDA approval for the treatment of MS, to Q2 2024.

**FIGURE 1:**
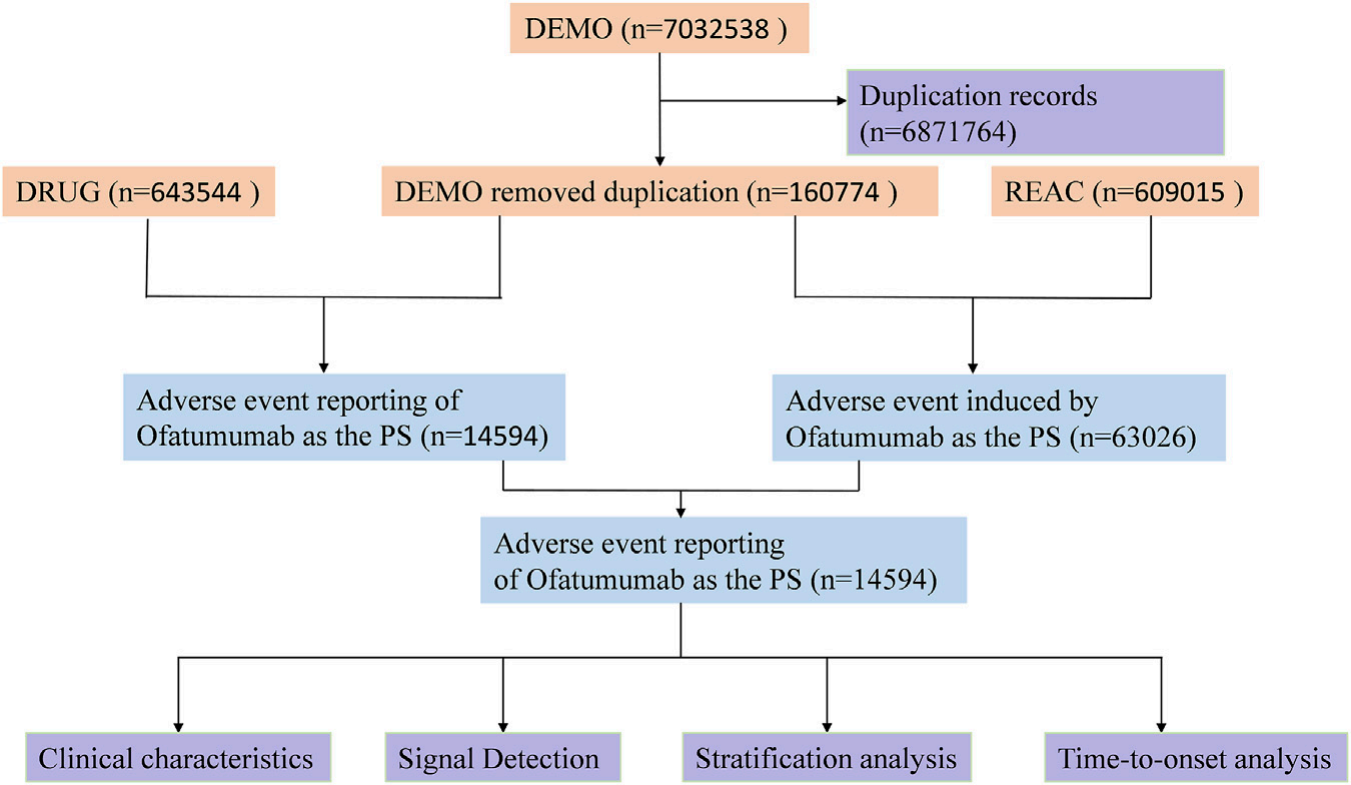
Flowchart illustrating the adverse event analysis process for Ofatumumab using the FDA Adverse Event Reporting System database.

To ensure data quality, duplicate reports were handled according to FDA-recommended practices. For reports with identical CASEIDs, the record with the most recent FDA receipt date (FDA_DT) was retained; if both the CASEID and FDA_DT were the same, the report with the highest PRIMARYID (unique report identifier) was selected. All adverse event descriptions were harmonized with the MedDRA dictionary to maintain uniformity in the data (Doan, 2000). Figure 1 provides an illustration of the study design workflow.

To minimize confounding factors, adverse event reports where ofatumumab was co-administered with medications commonly used for MS symptom management (e.g., gabapentin, baclofen, duloxetine) were excluded. This step ensured a more precise evaluation of the independent adverse event profile of ofatumumab. However, as a spontaneous reporting system, FAERS does not always capture complete information on comorbidities, co-medications, or patient history, which remains a limitation of this study.

### 2.2 Statistical analysis

Descriptive statistical techniques were utilized to examine the characteristics of adverse event reports related to ofatumumab. Four disproportionality assessment methods were applied to identify potential safety signals: Reporting Odds Ratio (ROR) (Rothman et al., 2004), Proportional Reporting Ratio (PRR) (Evans et al., 2001), Bayesian Confidence Propagation Neural Network (BCPNN) (Ang et al., 2016), and Multi-item Gamma Poisson Shrinker (MGPS) (Rivkees and Szarfman, 2010). These methods utilize a 2 × 2 contingency table structure to assess the relationship between the drug and AEs (Tubert and Begaud, 1991) (detailed in Supplementary Table S1).

The use of multiple disproportionality methods ensures robust signal detection, as each method has unique strengths in detecting safety signals. ROR and PRR are widely used for their simplicity, while BCPNN and MGPS incorporate Bayesian and probabilistic frameworks, respectively, to account for variability in reporting frequencies. Signal detection thresholds followed established criteria as detailed in Supplementary Table S2: ROR: lower limit of 95% CI > 1, N ≥ 3; PRR: PRR ≥ 2, χ^2^ ≥ 4, N ≥ 3; BCPNN: IC025 > 0; MGPS: EBGM05 > 2. Any adverse event that reached a positive signal threshold in at least one detection method was considered a potential safety signal. These thresholds were selected to balance sensitivity and specificity in identifying potential safety signals relevant to clinical practice.

### 2.3 Analysis of adverse event onset time

To gain deeper insight into the temporal characteristics of AEs, this study utilized the Weibull distribution model to analyze the onset time of AEs associated with ofatumumab. The Weibull distribution was chosen for its flexibility in modeling time-to-event data, allowing for the identification of both early (initial failure) and late (wear-out failure) risk patterns. Its shape parameter (β) provides insights into the nature of risk over time: β < 1 suggests decreasing risk, β = 1 indicates constant risk, and β > 1 reflects increasing risk. For ofatumumab, this model is particularly useful in pinpointing the clustering of adverse events during the early treatment phase, enabling clinicians to implement targeted monitoring strategies. By identifying high-risk periods, the findings can guide interventions aimed at mitigating adverse events and improving patient outcomes (Ogura and Shiraishi, 2022).

### 2.4 Statistical analysis tools

Data processing and analysis were performed with R software (version 4.3.3), maintaining a significance threshold of p < 0.05. The results included corresponding 95% confidence intervals to ensure statistical reliability.

## 3 Results

### 3.1 Descriptive analysis

A total of 14,594 adverse event reports associated with ofatumumab were included in this study. In terms of gender distribution, female patients accounted for the majority, with 10,707 reports (73.4%), while male patients contributed 3,216 reports (22.0%). Regarding age distribution, 46.1% of reports were from patients aged 18 to 65, whereas the proportion of reports from patients over 65 was relatively lower. The primary source of reports was consumers (CN), comprising 82.8%, followed by submissions from healthcare professionals (HP), physicians (MD), and pharmacists (PH). Geographically, the United States accounted for 79.8% of the reports, followed by the United Kingdom and Canada. Detailed descriptive statistics are provided in Table 1.

**TABLE 1:** Clinical characteristics of Ofatumumab adverse event reports from the FAERS database (Q3 2020 – Q2 2024).

Characteristics	Number of cases (Proportion of cases)
Number of events	N = 14,594
Gender	
F	10,707 (73.4%)
M	3,216 (22.0%)
Missing	671 (4.6%)
Age	
<18	11 (0.1%)
>85	2 (0.0%)
18∼65	6,723 (46.1%)
66∼85	346 (2.4%)
Missing	7,512 (51.5%)
Reporter	
CN	12,088 (82.8%)
HP	1,165 (8.0%)
MD	1,123 (7.7%)
PH	124 (0.8%)
Missing	94 (0.6%)
Top 5 Reported Countries	
US	11,641 (79.8%)
GB	436 (3.0%)
CA	312 (2.1%)
DE	202 (1.4%)
AU	197 (1.3%)

### 3.2 Distribution of AEs by system organ class (SOC)

**FIGURE 2:**
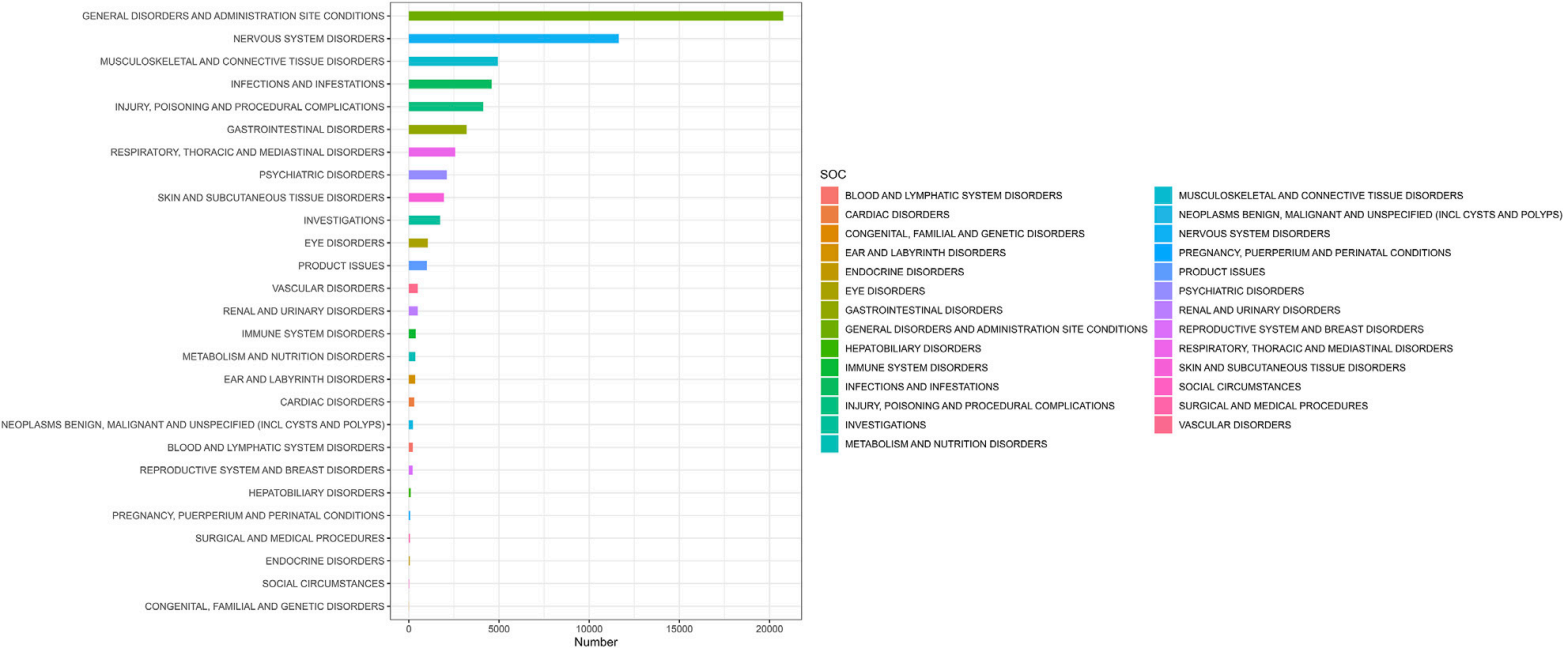
Proportion of adverse events by system organ class for Ofatumumab, showing the relative proportions across 27 SOC categories.

AEs associated with ofatumumab were reported across 27 SOC categories, encompassing systems such as general disorders and administration site conditions, infections and infestations, nervous system disorders, psychiatric disorders, respiratory, thoracic and mediastinal disorders, skin and subcutaneous tissue disorders, blood and lymphatic system disorders, and musculoskeletal and connective tissue disorders, among others. The distribution of AEs within these SOC categories is shown in Figure 2. Despite the broad range of organ systems affected, the assessment of signal strength using ROR, PRR, EBGM, and BCPNN indicated that many SOC categories did not demonstrate statistically significant associations with ofatumumab, failing to meet the standards for positive signal identification (as detailed in Table 2).

**TABLE 2:** Signal strength of ofatumumab AEs across System Organ Classes (SOC) in the FAERS database, identifying statistically significant safety signals.

System organ class (SOC)	Case numbers	ROR (95%Cl)	PRR (χ^2^)	EBGM(EBGM05)	IC(IC025)
Infections and infestations	4,597	0.73 (0.71–0.76)	0.75 (376.81)	0.77 (0.75)	−0.37 (−0.42)
Blood and lymphatic system disorders	223	0.39 (0.34–0.44)	0.39 (207.24)	0.41 (0.37)	−1.27 (−1.47)
Investigations	1,735	0.42 (0.4–0.45)	0.44 (1,252.73)	0.47 (0.45)	−1.1 (−1.17)
Immune system disorders	383	0.55 (0.5–0.61)	0.56 (128.61)	0.58 (0.53)	−0.78 (−0.93)
Skin and subcutaneous tissue disorders	1,943	0.81 (0.78–0.85)	0.82 (72.28)	0.84 (0.8)	−0.26 (−0.33)
Neoplasms benign, malignant and unspecified (incl cysts and polyps)	226	0.22 (0.19–0.25)	0.22 (621.22)	0.24 (0.21)	−2.07 (−2.26)
Reproductive system and breast disorders	213	0.65 (0.57–0.75)	0.66 (36.1)	0.68 (0.6)	−0.56 (−0.76)
General disorders and administration site conditions*	20,754	2.57 (2.53–2.62)	2.05 (11,036.62)	1.85 (1.82)	0.89 (0.87)
Cardiac disorders	303	0.38 (0.34–0.42)	0.38 (295.23)	0.41 (0.37)	−1.3 (−1.47)
Nervous system disorders	11,638	0.99 (0.97–1.01)	0.99 (1.76)	0.99 (0.97)	−0.02 (−0.04)
Respiratory, thoracic and mediastinal disorders*	2,569	1.28 (1.22–1.33)	1.26 (127.84)	1.23 (1.19)	0.3 (0.24)
Hepatobiliary disorders	100	0.3 (0.25–0.37)	0.31 (153.59)	0.33 (0.28)	−1.6 (−1.9)
Gastrointestinal disorders	3,201	0.75 (0.72–0.78)	0.76 (228.27)	0.78 (0.76)	−0.35 (−0.41)
Musculoskeletal and connective tissue disorders*	4,940	1.3 (1.26–1.34)	1.28 (274.01)	1.24 (1.21)	0.31 (0.27)
Injury, poisoning and procedural complications	4,123	0.75 (0.73–0.78)	0.77 (290.28)	0.79 (0.77)	−0.35 (−0.39)
Metabolism and nutrition disorders	362	0.5 (0.45–0.55)	0.5 (173.18)	0.53 (0.48)	−0.92 (−1.08)
Eye disorders	1,055	0.72 (0.68–0.77)	0.73 (101.37)	0.75 (0.71)	−0.42 (−0.51)
Psychiatric disorders	2,104	0.74 (0.7–0.77)	0.75 (176.49)	0.77 (0.74)	−0.39 (−0.45)
Ear and labyrinth disorders	350	0.9 (0.81–1.01)	0.9 (3.27)	0.91 (0.83)	−0.13 (−0.29)
Product issues*	999	4.57 (4.23–4.94)	4.51 (1805.5)	3.31 (3.1)	1.73 (1.62)
Vascular disorders	492	0.3 (0.28–0.33)	0.31 (747.52)	0.33 (0.31)	−1.58 (−1.72)
Social circumstances	23	0.11 (0.07–0.17)	0.11 (161.71)	0.12 (0.09)	−3.03 (−3.62)
Renal and urinary disorders	492	0.47 (0.43–0.52)	0.48 (269.47)	0.51 (0.47)	−0.98 (−1.12)
Endocrine disorders	49	0.34 (0.26–0.45)	0.34 (59.69)	0.37 (0.29)	−1.44 (−1.86)
Pregnancy, puerperium and perinatal conditions	77	0.22 (0.18–0.28)	0.22 (206.54)	0.24 (0.2)	−2.05 (−2.38)
Surgical and medical procedures	61	0.12 (0.09–0.16)	0.12 (385.44)	0.13 (0.11)	−2.91 (−3.27)
NANA	4	0.08 (0.03–0.22)	0.08 (40.46)	0.09 (0.04)	−3.46 (−4.75)
Congenital, familial and genetic disorders	10	0.17 (0.09–0.32)	0.17 (40.05)	0.19 (0.11)	−2.43 (−3.31)

Abbreviation: Asterisks (*) indicate statistically significant signals in algorithm; ROR, reporting odds ratio; PRR, proportional reporting ratio; EBGM, empirical Bayesian geometric mean; EBGM05, the lower limit of the 95% CI, of EBGM; IC, information component; IC025, the lower limit of the 95% CI, of the IC; CI, confidence interval; AEs, adverse events.

### 3.3 Distribution of AEs at the preferred term (PT) level

**FIGURE 3:**
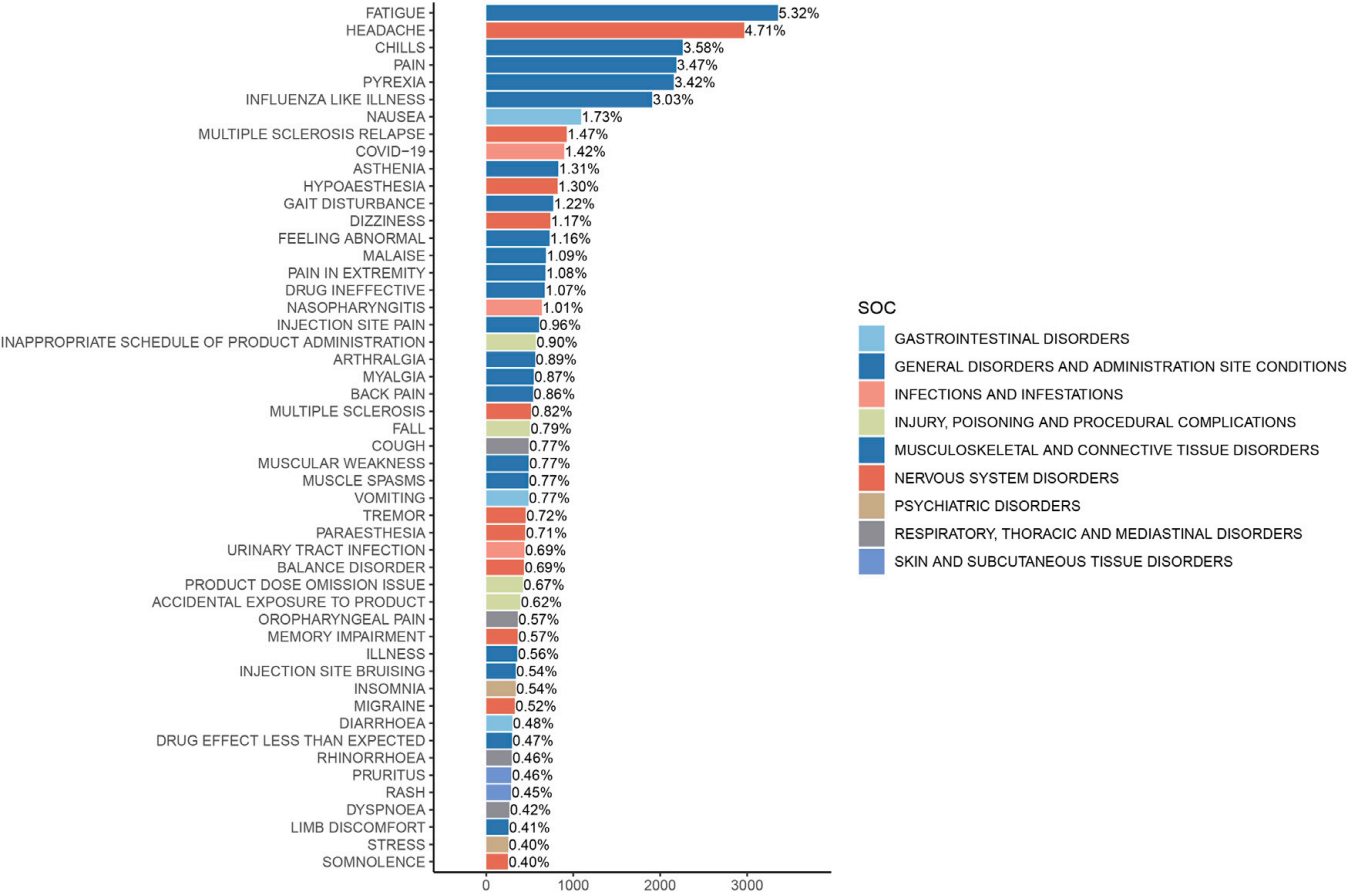
Top 50 adverse events by frequency at the PT level for Ofatumumab, highlighting both known reactions and newly observed events.

**FIGURE 4:**
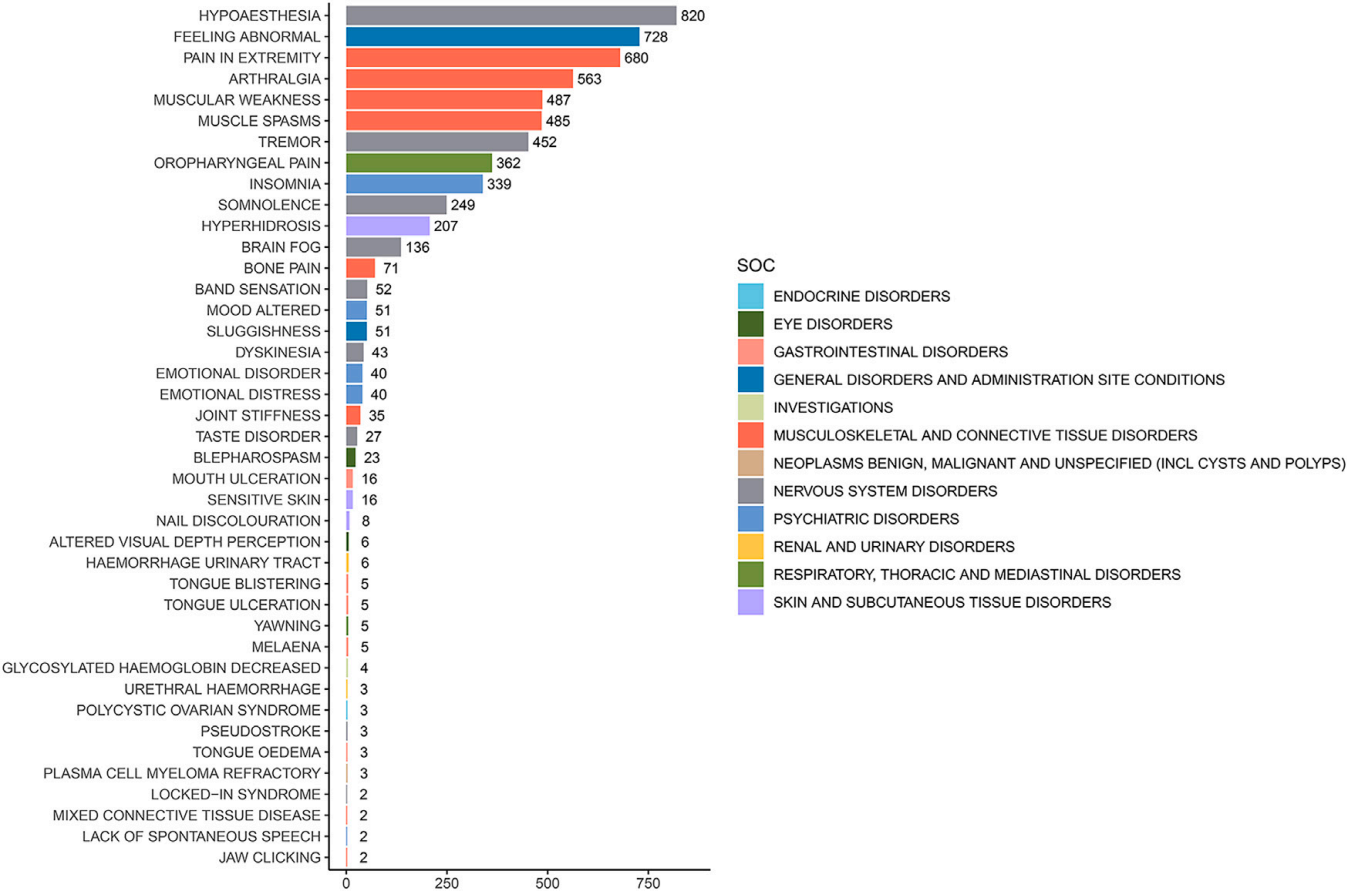
Potential off-label adverse events at the PT level for Ofatumumab.

The analysis of AEs at the PT level involved ranking the frequency of AEs linked to ofatumumab and assessing positive signals. The top 50 most frequently reported AEs meeting the criteria for positive signals are presented in Figure 3 and Table 3. The analysis confirmed known AEs listed on the drug label, such as upper respiratory tract infections, urinary tract infections, headache, injection-related reactions, hypersensitivity, and decreased immunoglobulin levels. Additionally, the study identified potential AEs not specified on the drug label (Figure 4), including hypoaesthesia, feeling abnormal, arthralgia, muscular weakness, muscle spasms, tremor, insomnia, somnolence, hyperhidrosis, brain fog, bone pain, band sensation, mood altered, sluggishness, dyskinesia, emotional disorder, joint stiffness, taste disorder, and blepharospasm. Detailed information on these PTs can be found in Supplementary Table S3.

**TABLE 3:** Top 50 frequency of adverse events at the PT level for Ofatumumab, highlighting both known and potential off-label events.

PT	Case numbers	ROR (95%CI)	PRR (χ^2^)	EBGM(EBGM05)	IC(IC025)
Fatigue*	3,355	2.13 (2.04–2.21)	2.07 (1,532.37)	1.86 (1.8)	0.9 (0.84)
Headache*	2,967	3.03 (2.9–3.16)	2.93 (2,880.58)	2.44 (2.36)	1.29 (1.23)
Chills*	2,259	14.32 (13.39–15.31)	13.84 (10,422.21)	5.94 (5.62)	2.57 (2.49)
Pain*	2,187	3.21 (3.06–3.37)	3.13 (2,366.43)	2.57 (2.46)	1.36 (1.29)
Pyrexia*	2,156	6.16 (5.83–6.51)	5.98 (5,339.96)	3.95 (3.77)	1.98 (1.91)
Influenza like illness*	1,908	8.45 (7.93–9)	8.22 (6,250.84)	4.71 (4.46)	2.23 (2.15)
Nausea*	1,090	1.63 (1.52–1.74)	1.62 (218.22)	1.52 (1.44)	0.6 (0.51)
Multiple sclerosis relapse	925	0.54 (0.5–0.58)	0.54 (339.35)	0.57 (0.54)	−0.81 (−0.9)
COVID-19	896	0.56 (0.52–0.6)	0.57 (288.57)	0.59 (0.56)	−0.76 (−0.86)
Asthenia*	828	1.38 (1.28–1.49)	1.38 (74.84)	1.33 (1.25)	0.41 (0.3)
Hypoaesthesia*	820	1.32 (1.22–1.42)	1.31 (53.26)	1.27 (1.19)	0.35 (0.24)
Gait disturbance	770	1.02 (0.95–1.1)	1.02 (0.29)	1.02 (0.96)	0.03 (−0.08)
Dizziness*	737	1.21 (1.12–1.3)	1.2 (22.35)	1.18 (1.1)	0.24 (0.12)
Feeling abnormal*	728	2.07 (1.91–2.25)	2.06 (323.22)	1.86 (1.74)	0.89 (0.78)
Malaise*	685	1.74 (1.6–1.89)	1.73 (178.76)	1.61 (1.5)	0.69 (0.57)
Pain in extremity*	680	1.43 (1.32–1.55)	1.42 (74.1)	1.36 (1.27)	0.45 (0.33)
Drug ineffective	672	0.84 (0.77–0.9)	0.84 (19.69)	0.85 (0.8)	−0.23 (−0.35)
Nasopharyngitis*	639	1.89 (1.73–2.06)	1.88 (217.44)	1.72 (1.6)	0.78 (0.66)
Injection site pain*	607	3.66 (3.33–4.02)	3.63 (819.16)	2.86 (2.64)	1.51 (1.38)
Inappropriate schedule of product administration*	569	3.62 (3.28–3.99)	3.6 (756.57)	2.84 (2.61)	1.5 (1.37)
Arthralgia*	563	1.83 (1.67–2.01)	1.83 (174.67)	1.68 (1.56)	0.75 (0.62)
Myalgia*	546	4.91 (4.42–5.45)	4.87 (1,077.87)	3.48 (3.18)	1.8 (1.65)
Back pain*	539	1.63 (1.48–1.79)	1.62 (109.01)	1.52 (1.41)	0.61 (0.47)
Multiple sclerosis	514	0.42 (0.38–0.46)	0.42 (392.79)	0.45 (0.42)	−1.15 (−1.28)
Fall	501	0.55 (0.5–0.6)	0.55 (176.8)	0.58 (0.53)	−0.8 (−0.93)
Cough*	487	1.64 (1.48–1.8)	1.63 (100.5)	1.53 (1.41)	0.61 (0.47)
Muscular weakness*	487	1.22 (1.11–1.35)	1.22 (17.3)	1.19 (1.1)	0.26 (0.12)
Muscle spasms*	485	1.58 (1.43–1.74)	1.57 (85.71)	1.48 (1.37)	0.57 (0.43)
Vomiting*	485	1.39 (1.26–1.53)	1.39 (45.06)	1.33 (1.23)	0.41 (0.27)
Tremor*	452	1.75 (1.58–1.94)	1.75 (120.93)	1.62 (1.49)	0.7 (0.55)
Paraesthesia	447	1.03 (0.93–1.13)	1.03 (0.28)	1.02 (0.94)	0.03 (−0.11)
Urinary tract infection	438	0.71 (0.64–0.78)	0.71 (48.29)	0.73 (0.67)	−0.45 (−0.59)
Balance disorder	433	0.93 (0.84–1.02)	0.93 (2.2)	0.93 (0.86)	−0.1 (−0.24)
Product dose omission issue	422	0.56 (0.51–0.62)	0.56 (136.31)	0.59 (0.54)	−0.76 (−0.91)
Accidental exposure to product*	390	82.91 (60.09–114.39)	82.4 (2,985.51)	8.74 (6.68)	3.13 (2.93)
Oropharyngeal pain*	362	2.49 (2.22–2.8)	2.48 (250.29)	2.15 (1.95)	1.11 (0.94)
Memory impairment	361	0.6 (0.54–0.67)	0.6 (90.14)	0.63 (0.57)	−0.67 (−0.83)
Illness*	356	1.35 (1.21–1.51)	1.35 (27.7)	1.3 (1.18)	0.38 (0.22)
Injection site bruising*	339	5.58 (4.86–6.39)	5.55 (772.06)	3.77 (3.37)	1.92 (1.73)
Insomnia*	339	1.19 (1.06–1.33)	1.19 (8.95)	1.17 (1.06)	0.22 (0.06)
Migraine*	328	1.77 (1.57–2)	1.77 (91.2)	1.64 (1.48)	0.71 (0.54)
Diarrhoea	300	0.54 (0.48–0.6)	0.54 (112.57)	0.57 (0.51)	−0.82 (−0.99)
Drug effect less than expected*	296	33.45 (26.03–42.99)	33.3 (1915.68)	7.67 (6.22)	2.94 (2.71)
Rhinorrhoea*	293	3.16 (2.76–3.61)	3.15 (315.09)	2.57 (2.3)	1.36 (1.17)
Pruritus	287	0.74 (0.66–0.84)	0.74 (23.43)	0.76 (0.69)	−0.39 (−0.56)
Rash	284	1.05 (0.93–1.18)	1.05 (0.53)	1.04 (0.94)	0.06 (−0.12)
Dyspnoea	266	0.74 (0.65–0.84)	0.74 (22.07)	0.76 (0.69)	−0.39 (−0.57)
Limb discomfort*	256	1.73 (1.51–1.98)	1.73 (65.88)	1.61 (1.44)	0.69 (0.49)
Stress	253	1.03 (0.91–1.18)	1.03 (0.22)	1.03 (0.92)	0.04 (−0.15)
Somnolence*	249	1.34 (1.18–1.54)	1.34 (18.96)	1.3 (1.16)	0.38 (0.18)

Abbreviation: Asterisks (*) indicate statistically significant signals in algorithm; ROR, reporting odds ratio; PRR, proportional reporting ratio; EBGM, empirical Bayesian geometric mean; EBGM05, the lower limit of the 95% CI, of EBGM; IC, information component; IC025, the lower limit of the 95% CI, of the IC; CI, confidence interval; PT, preferred term.

### 3.4 Subgroup analysis

**FIGURE 5:**
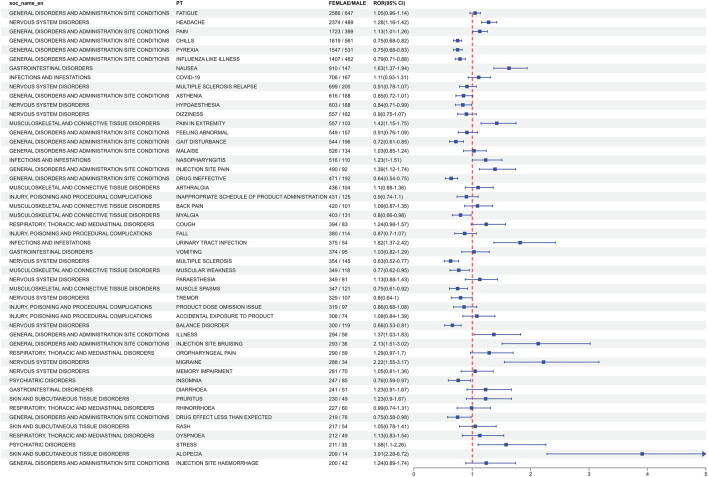
Gender-specific differences in the top 50 adverse events at The PT level for Ofatumumab, emphasizing the variation in event frequencies between males and females.

The subgroup analysis of AEs related to ofatumumab revealed gender-specific differences among the top 50 most common AEs meeting the criteria for positive signals. Male-specific events included hyperhidrosis, somnolence, nasopharyngitis, and shortened therapeutic response. In contrast, female patients more frequently reported migraine, injection site bruising, injection site hemorrhage, and pain in extremities (Figure 5). Detailed data can be found in Supplementary Tables S4, S5.

The age subgroup analysis indicated that the distribution of AEs was relatively consistent across all age groups, with fatigue, headache, chills, pyrexia, and dizziness being common positive signals in all cohorts. However, in the 18–65 age group, which represents the peak incidence period for MS, additional off-label positive signal AEs were identified, including muscle spasms, migraine, arthralgia, feeling abnormal, and hypoaesthesia. In the 45–65 age group, further off-label positive signals such as muscular weakness, tremor, and musculoskeletal stiffness were observed. Among individuals aged 65 and above, off-label positive signals included tremor, insomnia, and somnolence. Detailed data for these findings are provided in Supplementary Tables S6–S9.

### 3.5 Sensitivity analysis

In clinical practice, ofatumumab is often co-administered with medications such as gabapentin, baclofen, duloxetine, vitamin B, and vitamin D. To evaluate the independent adverse event profile of ofatumumab, this study excluded 2,645 reports involving these commonly co-administered drugs. When assessing only reports involving ofatumumab as a standalone treatment, persistent positive signal AEs included fatigue, headache, chills, pyrexia, dizziness, muscle spasms, tremor, insomnia, migraine, somnolence, and urinary tract infection. Detailed findings are provided in Supplementary Table S10.

### 3.6 Onset time and weibull distribution analysis of AEs

**FIGURE 6:**
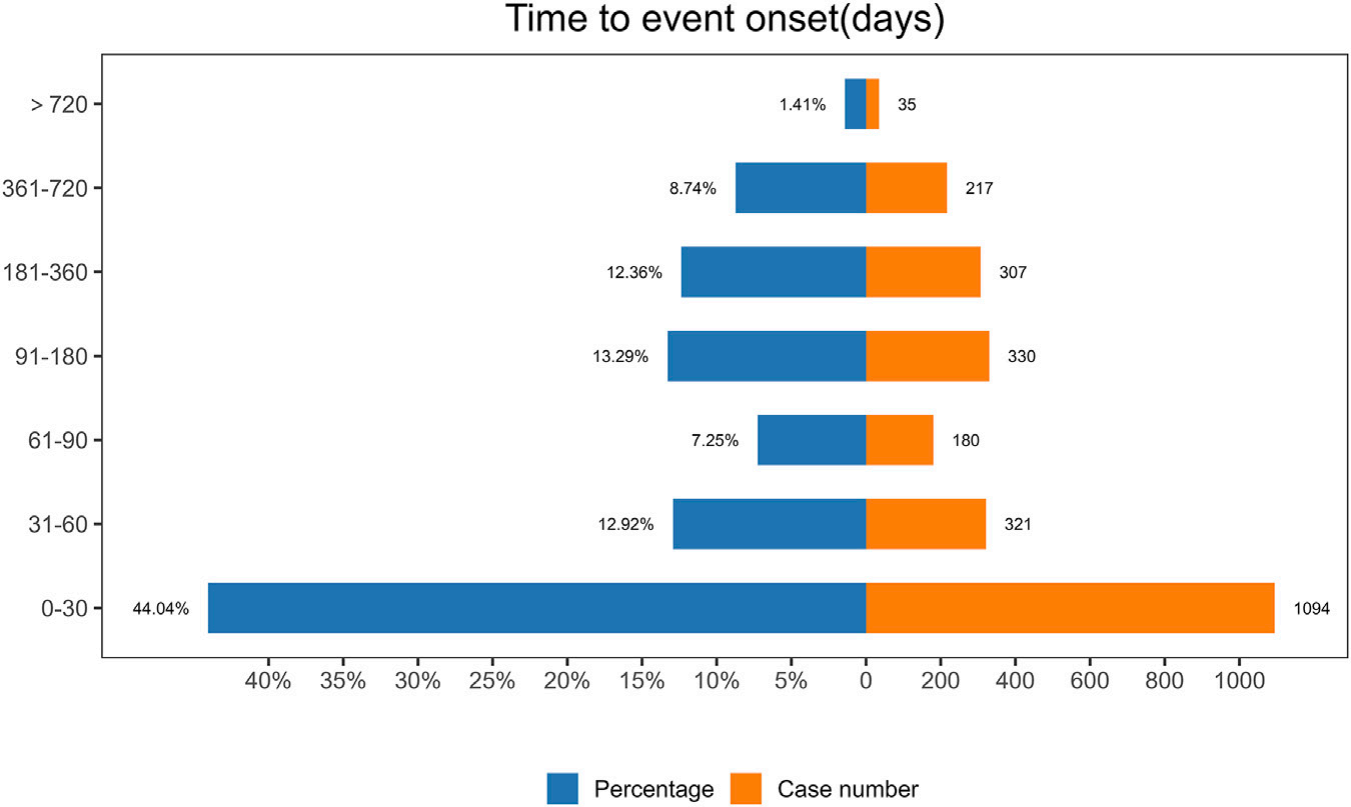
Onset time distribution of adverse events associated with Ofatumumab, illustrating the clustering of events within the first month of treatment.

**FIGURE 7:**
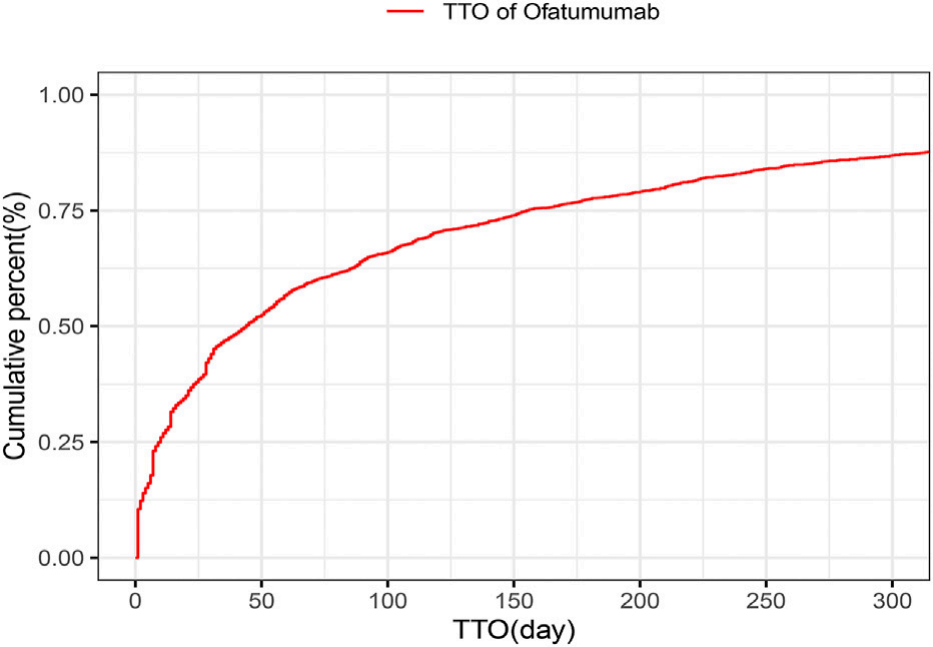
Cumulative incidence curve of adverse events for Ofatumumab treatment, emphasizing the early risk phase.

The analysis of the onset time of AEs related to ofatumumab revealed that most AEs took place during the first month of treatment (Figure 6). The cumulative incidence curve is shown in Figure 7. The Weibull distribution assessment revealed an initial failure pattern for the drug, suggesting a higher risk of adverse events during the initial treatment phase. The results showed that the median time to onset (TTO) for AEs was 44 days (IQR: 10–155 days). The scale parameter (α) of the Weibull distribution was 86.8 (95% CI: 80.9–92.6), and the shape parameter (β) was 0.617 (95% CI: 0.598–0.635). Detailed parameters are provided in Table 4.

**TABLE 4:** Time to onset of Ofatumumab-associated adverse events and Weibull distribution analysis.

Drug	TTO (days)		Weibull distribution		
Ofatumumab	Case reports	Median(d) (IQR)	Scale parameter: α(95%CI)	Shape parameter: β(95%CI)	Type
	2,484	44 (10,155)	86.8 (80.9–92.6)	0.617 (0.598–0.635)	Early failure

Abbreviation: TTO, time to onset; CI, confidence interval; IQR, interquartile range.

## 4 Discussion

This research, utilizing real-world data from the FAERS database, confirmed known adverse reactions to ofatumumab, including injection site reactions, upper respiratory tract infections, headache, and decreased levels of immunoglobulin M (IgM). Additionally, we identified, for the first time, potential off-label adverse reactions such as brain fog, muscle spasms, and mood alterations. These new findings expand the safety profile of ofatumumab and provide clinicians with more comprehensive information for risk management.

This study confirmed on-label adverse reactions aligning with results reported in various clinical trials. For instance, in the ASCLEPIOS I and II trials, injection-related reactions occurred in 16.1%–24.1% of patients, presenting as mild to moderate symptoms such as fever, headache, and myalgia, primarily during the initial injection and decreasing with subsequent administrations (Carlson et al., 2024). Additionally, the infection rate in the ofatumumab treatment groups ranged from 49.2% to 53.8%, with most cases involving upper respiratory tract and urinary tract infections, while the incidence of serious infections was 2.5%–2.9% (Carlson et al., 2024). In the ALITHIOS open-label extension study, long-term safety data for ofatumumab indicated that 24.7% of patients experienced mild to moderate injection-related reactions, and 58.35% reported infections, with a serious infection rate of 4.01% (Hauser et al., 2023). Our findings align with these results, underscoring the need for clinicians to closely monitor for injection-related reactions and infections, especially during the initial treatment phase.

The study found that ofatumumab treatment for relapsing MS may lead to various off-label adverse reactions. Most of these reactions were observed in the nervous system, gastrointestinal system, skin and subcutaneous tissue, musculoskeletal and connective tissue, and psychiatric systems, potentially impacting patients’ quality of life to varying extents. However, the exact mechanisms remain unclear and are hypothesized to be related to B-cell depletion-induced immunosuppression, cytokine imbalance, and potential immune responses (Krajnc et al., 2022).

In terms of the nervous and psychiatric systems, off-label AEs potentially associated with ofatumumab treatment include hypoaesthesia, pseudostroke, locked-in syndrome, movement disorders, tremor, somnolence, brain fog, band sensation, taste disorder, insomnia, mood alterations, and emotional disorders. Research indicates that B cells contribute substantially to the inflammatory environment of the CNS in MS patients by releasing pro-inflammatory factors, like IL-6 and TNF-α, and reducing anti-inflammatory factors, like IL-10, thereby sustaining a state of chronic inflammation (Delgado et al., 2024). Moreover, the aggregation of B cells around the meninges and perivascular regions is closely associated with inflammatory demyelinating lesions. Particularly during the chronic stage of the disease, these B cell clusters form follicle-like structures, which further exacerbate neuroinflammation and the demyelination process (Haghikia et al., 2024). Ofatumumab targets CD20 to achieve significant B-cell depletion, reducing inflammatory responses and supporting its effectiveness in treating MS. However, extensive B-cell depletion may disrupt immune regulation in the CNS, particularly due to the loss of specific regulatory B-cell types (Bouaziz et al., 2008; Li et al., 2018). This disruption can lead to immune imbalances, potentially causing neurological and psychiatric adverse effects (Wu et al., 2022). For example, the loss of B cell-mediated immune regulatory support may cause the CNS to overreact to minor inflammatory triggers, resulting in symptoms such as somnolence, tremor, and brain fog (Ran et al., 2020). Moreover, certain pro-inflammatory factors released by B cells, such as IL-6, play an indirect role in mood and cognitive regulation. As a result, B-cell depletion caused by ofatumumab may disrupt this balance, possibly leading to mood changes and sleep disturbances (De Sèze et al., 2023; Krajnc et al., 2022). For this reason, it is essential for clinicians to monitor patients’ neurological and psychiatric symptoms closely, especially in the early stages of treatment, to detect and manage any adverse reactions. When needed, psychological support or medication should be offered to help patients handle these side effects and preserve their quality of life.

The study also found that ofatumumab treatment for MS may lead to a range of off-label AEs affecting the gastrointestinal, skin, and mucosal systems. B cells serve an essential function in immune regulation within the gastrointestinal tract and skin mucosa, forming a barrier against external pathogens (Castro-Dopico et al., 2020; Zouali, 2021). However, CD20-targeted B-cell depletion can disrupt immune regulation, potentially weakening mucosal barrier function. Consequently, this disruption may lead to adverse effects, including mucosal irritation and increased skin sensitivity (Delgado et al., 2024; Krajnc et al., 2022). For this reason, close monitoring of gastrointestinal and skin symptoms is advised, along with personalized interventions to help reduce the impact of adverse reactions on patients’ quality of life.

Additionally, AEs related to the musculoskeletal and connective tissue systems commonly manifest as symptoms such as pain in the extremities, arthralgia, muscular weakness, muscle spasms, and bone pain. Notably, similar to their role in the nervous and mucosal systems, B cells also play a significant role in regulating local inflammation in joint and muscle tissues (Carter et al., 2012). B-cell depletion induced by ofatumumab may lead to local immune dysregulation, potentially resulting in symptoms associated with the musculoskeletal system. It is recommended that patients exhibiting musculoskeletal symptoms receive physical therapy or pharmacological interventions to alleviate symptoms and enhance their capacity for daily activities.

In summary, these off-label AEs may stem from the complex mechanisms of action of ofatumumab in neuroregulation, immune modulation, and emotional regulation. Given that some of these mechanisms remain unclear, it is essential to enhance monitoring in clinical practice. Additionally, individualized management strategies should be developed for both the initial treatment phase and long-term care to mitigate the impact on patients’ quality of life.

Subgroup analysis revealed that among male patients receiving ofatumumab treatment, common AEs included hyperhidrosis, somnolence, and nasopharyngitis, while female patients more frequently reported migraine, injection site bruising, and pain in the extremities. Across different age groups, symptoms such as fatigue and headache are commonly observed. However, patients aged 18 to 65 are more likely to experience muscle spasms and migraines, while individuals over 65 are more prone to tremors and insomnia. Based on these findings, clinical practice should enhance monitoring of specific AEs according to gender and age characteristics to improve medication safety and patient comfort. The observed gender differences in adverse events may be attributed to biological and hormonal factors. Hyperhidrosis, more commonly reported in males, could be linked to higher sweat gland density and androgen activity, which are known to influence sweat production (Kunkeler and Hodgins, 1994). Conversely, migraines, more frequently reported in females, are associated with hormonal fluctuations, particularly estrogen, which plays a role in trigeminal nerve sensitivity and vascular regulation (Chai et al., 2014; Nappi et al., 2022). These findings highlight the need for gender-specific monitoring and management strategies to mitigate these adverse events effectively. Based on these findings, clinical practice should enhance monitoring of specific AEs according to gender and age characteristics to improve medication safety and patient comfort.

Notably, the sensitivity analysis validated the independent adverse event profile of ofatumumab in the absence of co-administered common medications. The results indicated that adverse reactions such as fatigue, headache, chills, pyrexia, dizziness, muscle spasms, tremor, insomnia, migraine, somnolence, and urinary tract infection remained persistent positive signals. The persistent presence of these AEs suggests that they may represent independent side effects of ofatumumab treatment rather than being attributed to co-administered medications. Although these symptoms are mostly non-fatal adverse reactions, they can significantly impact patients’ quality of life and treatment adherence; therefore, they should be addressed in clinical management.

This study also performed a time-based analysis of AEs and used the Weibull distribution to forecast when these events might occur, providing an effective temporal framework for monitoring adverse reactions. The results show that adverse events related to ofatumumab are primarily concentrated in the early stages of treatment, particularly within the first month. This clustering of adverse events during the first month is likely driven by both pharmacokinetic and immunological mechanisms. Pharmacokinetically, the initial doses of ofatumumab rapidly deplete circulating B cells, resulting in transient immune perturbations. Immunologically, B-cell depletion can lead to a temporary imbalance in cytokine levels, including increased pro-inflammatory cytokines, which may account for early onset adverse reactions such as fatigue, chills, and injection site reactions. These mechanisms highlight the importance of understanding both the pharmacokinetic and immunological dynamics of ofatumumab to better predict and manage adverse events during this critical period (Smith et al., 2017; Theil et al., 2019). This early pattern underscores the importance of vigilant monitoring during the initial treatment period to promptly identify and manage any adverse reactions, thereby enhancing patient safety and treatment outcomes. Specific strategies for monitoring include comprehensive baseline assessments of immune status, neurological function, and psychiatric history prior to initiating treatment; frequent clinical follow-ups during the first 1–3 months to monitor for early adverse reactions; and rapid-response protocols for addressing identified issues, such as symptomatic treatment for injection site reactions or psychological support for mood disturbances. Additionally, educating patients about potential early adverse effects and encouraging timely reporting of symptoms can further optimize treatment adherence and safety. Together, these measures aim to mitigate risks during this critical period and improve overall patient outcomes.

The study also identified discrepancies in certain system organ classes (SOC) where no statistically significant signals were observed, despite high case numbers. This discrepancy is likely due to the nature of disproportionality methods, such as ROR and PRR, which evaluate the reporting rates of adverse events relative to the entire FAERS database rather than raw frequencies. High baseline reporting rates for certain SOCs, such as infections or general disorders, may dilute the signal strength of specific drugs, as these events are commonly reported across various drugs and conditions. For example, infections are frequently reported in the FAERS database, and this high baseline may reduce the likelihood of detecting significant signals for ofatumumab within this category. This finding underscores the importance of interpreting disproportionality results within the broader context of background reporting trends and highlights the limitations of relying solely on absolute case counts. Future studies could benefit from alternative analytical approaches, such as machine learning-based methods or integrated data sources, to address these limitations and enhance signal detection sensitivity.

Building on this, a comparative analysis of adverse event profiles between ofatumumab and other monoclonal antibody therapies, such as ocrelizumab, represents a promising direction for future research. By leveraging real-world data from the FAERS database, this work could identify differences in safety signals that may inform clinical decision-making and support the development of personalized treatment strategies for patients with multiple sclerosis. Such studies could also provide insights into whether observed safety profiles are drug-specific or reflect broader class effects associated with CD20-targeted therapies.

It is important to highlight that this study has some limitations. First, as a source of data that relies on spontaneous reporting, the FAERS database may be subject to information gaps and biases, particularly regarding details such as medication exposure, symptom severity, and patient history. Such incompleteness may impact the accuracy of the analytical results. Additionally, although the dataset encompasses multiple reports, the sample size remains relatively limited, requiring further validation with larger-scale data support.

Furthermore, the FAERS database may contain duplicate or misclassified reports, which can confound signal detection efforts and introduce inaccuracies in disproportionality analysis. Underreporting is another significant challenge, as many mild or unrecognized adverse events may go unreported, leading to an incomplete picture of the drug’s safety profile. Selection bias also plays a role, as more severe or well-known adverse events are more likely to be reported, skewing the results.

The data in the FAERS database is primarily derived from the United States, which may introduce regional bias and restrict the applicability of the study findings to other regions. Lastly, this study employed disproportionality analysis to identify adverse event signals, however, this method is limited in establishing causal relationships between the drug and AEs.

To address these limitations, future studies should consider integrating FAERS data with other real-world evidence sources, such as electronic health records, claims databases, or clinical trial data, to provide a more comprehensive and reliable assessment of drug safety.

As a result, future research will still need to rely on prospective studies to validate these potential associations.

## 5 Conclusion

This research conducted a systematic analysis of the real-world AEs associated with ofatumumab using data from the FAERS database since 2020. The findings confirmed previously known adverse reactions and also revealed potential off-label effects, including brain fog and muscle spasms. Overall, these insights offer valuable safety information for clinical application. Subgroup analysis revealed differences in AEs based on gender and age, with males being more prone to symptoms such as hyperhidrosis, while elderly patients were more likely to experience neurological adverse reactions. The sensitivity analysis further validated the independent adverse reaction profile of ofatumumab, while the temporal analysis indicated that AEs were primarily concentrated in the initial stages of treatment.

To translate these findings into clinical practice, it is essential to enhance monitoring strategies during the initial phase of treatment, particularly within the first month, by conducting comprehensive baseline assessments, scheduling frequent follow-ups, and implementing early intervention protocols tailored to high-risk subgroups such as elderly patients and those with pre-existing neurological conditions. Additionally, educating patients about potential early adverse effects and encouraging timely reporting of symptoms can further improve safety and adherence.

These results also underscore the critical need for prospective studies to validate these findings and explore the underlying mechanisms of off-label adverse events, particularly those related to B-cell depletion-induced immune dysregulation. By addressing these gaps, researchers and clinicians can better optimize treatment strategies and enhance outcomes for patients with multiple sclerosis.

## Data Availability

The original contributions presented in the study are included in the article/Supplementary Material, further inquiries can be directed to the corresponding author.
