# Supplementary material for: Real-world pharmacovigilance of ofatumumab in multiple sclerosis: a comprehensive FAERS data analysis
Source: Front Pharmacol. 2025 Jan 23;15:1521726. doi: 10.3389/fphar.2024.1521726 (PMC11799251; doi:10.3389/fphar.2024.1521726)
Supplement: Supplementary file 1 [file Table1.docx]

Supplementary Material

Supplementary Tables

Supplementary Table S1: Two-by-two contingency table for disproportionality analyses.

|  | Target AEs | Other AEs | Total |
| --- | --- | --- | --- |
| Ofatumumab | a | b | a+b |
| Other drugs | c | d | c+d |
| Total | a+c | b+d | a+b+c+d |

Abbreviation: AEs, adverse events; a, number of reports containing both the target drug and target adverse drug reaction; b, number of reports containing other adverse drug reaction of the target drug; c, number of reports containing the target adverse drug reaction of other drugs; d, number of reports containing other drugs and other adverse drug reactions.

Supplementary Table S2: Four major algorithms used for signal detection.

| Algorithms | Equation | Criteria |
| --- | --- | --- |
| ROR | ROR=ad/b/c | lower limit of 95% CI>1, N≥3 |
|  | 95%CI=e^ln(ROR)±1.96(1/a+1/b+1/c+1/d)^0.5^ |  |
| PRR | PRR=a(c+d)/c/(a+b) | PRR≥2, χ^2^≥4, N≥3 |
|  | χ^2^=[(ad-bc)^2](a+b+c+d)/[(a+b)(c+d)(a+c)(b+d)] |  |
| BCPNN | IC=log_2_a(a+b+c+d)(a+c)(a+b) | IC025>0 |
|  | 95%CI= E(IC) ± 2V(IC)^0.5 |  |
| MGPS | EBGM=a(a+b+c+d)/(a+c)/(a+b) | EBGM05>2 |
|  | 95%CI=e^ln(EBGM)±1.96(1/a+1/b+1/c+1/d)^0.5^ |  |

Abbreviation: a, number of reports containing both the target drug and target adverse drug reaction; b, number of reports containing other adverse drug reaction of the target drug; c, number of reports containing the target adverse drug reaction of other drugs; d, number of reports containing other drugs and other adverse drug reactions. 95%CI, 95% confidence interval; N, the number of reports; χ2, chi-squared; IC, information component; IC025, the lower limit of 95% CI of the IC; E(IC), the IC expectations; V(IC), the variance of IC; EBGM, empirical Bayesian geometric mean; EBGM05, the lower limit of 95% CI of EBGM.

Supplementary Table S3: Potential adverse effects at the preferred term (PT) level not listed on the Ofatumumab label from FAERS data.

| PT | Case numbers | ROR(95%Cl) | PRR(χ^2^) | EBGM  (EBGM05) | IC(IC025) |
| --- | --- | --- | --- | --- | --- |
| Hypoaesthesia | 820 | 1.32 ( 1.22 - 1.42 ) | 1.31 ( 53.26 ) | 1.27 ( 1.19 ) | 0.35 ( 0.24 ) |
| Feeling abnormal | 728 | 2.07 ( 1.91 - 2.25 ) | 2.06 ( 323.22 ) | 1.86 ( 1.74 ) | 0.89 ( 0.78 ) |
| Pain in extremity | 680 | 1.43 ( 1.32 - 1.55 ) | 1.42 ( 74.1 ) | 1.36 ( 1.27 ) | 0.45 ( 0.33 ) |
| Arthralgia | 563 | 1.83 ( 1.67 - 2.01 ) | 1.83 ( 174.67 ) | 1.68 ( 1.56 ) | 0.75 ( 0.62 ) |
| Muscular weakness | 487 | 1.22 ( 1.11 - 1.35 ) | 1.22 ( 17.3 ) | 1.19 ( 1.1 ) | 0.26 ( 0.12 ) |
| Muscle spasms | 485 | 1.58 ( 1.43 - 1.74 ) | 1.57 ( 85.71 ) | 1.48 ( 1.37 ) | 0.57 ( 0.43 ) |
| Tremor | 452 | 1.75 ( 1.58 - 1.94 ) | 1.75 ( 120.93 ) | 1.62 ( 1.49 ) | 0.7 ( 0.55 ) |
| Oropharyngeal pain | 362 | 2.49 ( 2.22 - 2.8 ) | 2.48 ( 250.29 ) | 2.15 ( 1.95 ) | 1.11 ( 0.94 ) |
| Insomnia | 339 | 1.19 ( 1.06 - 1.33 ) | 1.19 ( 8.95 ) | 1.17 ( 1.06 ) | 0.22 ( 0.06 ) |
| Somnolence | 249 | 1.34 ( 1.18 - 1.54 ) | 1.34 ( 18.96 ) | 1.3 ( 1.16 ) | 0.38 ( 0.18 ) |
| Hyperhidrosis | 207 | 2.1 ( 1.8 - 2.44 ) | 2.09 ( 95.55 ) | 1.88 ( 1.66 ) | 0.91 ( 0.69 ) |
| Brain fog | 136 | 5.13 ( 4.15 - 6.34 ) | 5.12 ( 283.71 ) | 3.59 ( 3.01 ) | 1.84 ( 1.56 ) |
| Bone pain | 71 | 1.95 ( 1.51 - 2.53 ) | 1.95 ( 26.94 ) | 1.78 ( 1.43 ) | 0.83 ( 0.46 ) |
| Band sensation | 52 | 1.75 ( 1.3 - 2.36 ) | 1.75 ( 13.99 ) | 1.63 ( 1.27 ) | 0.7 ( 0.27 ) |
| Mood altered | 51 | 2.28 ( 1.67 - 3.1 ) | 2.28 ( 28.95 ) | 2.01 ( 1.55 ) | 1.01 ( 0.57 ) |
| Sluggishness | 51 | 3.25 ( 2.36 - 4.48 ) | 3.25 ( 57.75 ) | 2.64 ( 2.01 ) | 1.4 ( 0.95 ) |
| Dyskinesia | 43 | 1.53 ( 1.1 - 2.11 ) | 1.53 ( 6.65 ) | 1.45 ( 1.1 ) | 0.53 ( 0.07 ) |
| Emotional disorder | 40 | 1.43 ( 1.02 - 2 ) | 1.43 ( 4.47 ) | 1.37 ( 1.04 ) | 0.45 ( -0.03 ) |
| Emotional distress | 40 | 1.41 ( 1.01 - 1.97 ) | 1.41 ( 4.08 ) | 1.35 ( 1.02 ) | 0.43 ( -0.05 ) |
| Joint stiffness | 35 | 1.66 ( 1.15 - 2.38 ) | 1.66 ( 7.65 ) | 1.55 ( 1.15 ) | 0.63 ( 0.11 ) |
| Taste disorder | 27 | 1.52 ( 1.01 - 2.29 ) | 1.52 ( 4.07 ) | 1.44 ( 1.02 ) | 0.53 ( -0.06 ) |
| Blepharospasm | 23 | 2.59 ( 1.62 - 4.12 ) | 2.59 ( 17.25 ) | 2.22 ( 1.51 ) | 1.15 ( 0.5 ) |
| Mouth ulceration | 16 | 1.93 ( 1.12 - 3.31 ) | 1.93 ( 5.82 ) | 1.76 ( 1.12 ) | 0.81 ( 0.05 ) |
| Sensitive skin | 16 | 1.95 ( 1.13 - 3.36 ) | 1.95 ( 6.07 ) | 1.78 ( 1.13 ) | 0.83 ( 0.07 ) |
| Nail discolouration | 8 | 2.89 ( 1.3 - 6.43 ) | 2.89 ( 7.4 ) | 2.42 ( 1.24 ) | 1.27 ( 0.19 ) |
| Altered visual depth perception | 6 | 2.74 ( 1.09 - 6.85 ) | 2.74 ( 5.02 ) | 2.32 ( 1.08 ) | 1.21 ( -0.02 ) |
| Haemorrhage urinary tract | 6 | 2.48 ( 1 - 6.13 ) | 2.48 ( 4.1 ) | 2.15 ( 1.01 ) | 1.1 ( -0.12 ) |
| Tongue blistering | 5 | 4.33 ( 1.48 - 12.67 ) | 4.33 ( 8.54 ) | 3.22 ( 1.31 ) | 1.69 ( 0.31 ) |
| Tongue ulceration | 5 | 4.33 ( 1.48 - 12.67 ) | 4.33 ( 8.54 ) | 3.22 ( 1.31 ) | 1.69 ( 0.31 ) |
| Yawning | 5 | 3.09 ( 1.11 - 8.59 ) | 3.09 ( 5.22 ) | 2.54 ( 1.08 ) | 1.35 ( 0 ) |
| Melaena | 5 | 2.71 ( 0.99 - 7.39 ) | 2.71 ( 4.1 ) | 2.3 ( 0.99 ) | 1.2 ( -0.13 ) |
| Glycosylated haemoglobin decreased | 4 | 5.78 ( 1.63 - 20.47 ) | 5.78 ( 9.48 ) | 3.87 ( 1.34 ) | 1.95 ( 0.39 ) |
| Urethral haemorrhage | 3 | 25.99 ( 2.7 - 249.87 ) | 25.99 ( 18.02 ) | 7.25 ( 1.09 ) | 2.86 ( 0.92 ) |
| Polycystic ovarian syndrome | 3 | 12.99 ( 2.17 - 77.77 ) | 12.99 ( 13.29 ) | 5.8 ( 1.3 ) | 2.54 ( 0.67 ) |
| Pseudostroke | 3 | 12.99 ( 2.17 - 77.77 ) | 12.99 ( 13.29 ) | 5.8 ( 1.3 ) | 2.54 ( 0.67 ) |
| Tongue oedema | 3 | 6.5 ( 1.45 - 29.03 ) | 6.5 ( 7.97 ) | 4.14 ( 1.18 ) | 2.05 ( 0.28 ) |
| Plasma cell myeloma refractory | 3 | 12.99 ( 2.17 - 77.77 ) | 12.99 ( 13.29 ) | 5.8 ( 1.3 ) | 2.54 ( 0.67 ) |
| Locked-in syndrome | 2 | 8.66 ( 1.22 - 61.5 ) | 8.66 ( 6.78 ) | 4.83 ( 0.94 ) | 2.27 ( 0.17 ) |
| Mixed connective tissue disease | 2 | 8.66 ( 1.22 - 61.5 ) | 8.66 ( 6.78 ) | 4.83 ( 0.94 ) | 2.27 ( 0.17 ) |
| Lack of spontaneous speech | 2 | 8.66 ( 1.22 - 61.5 ) | 8.66 ( 6.78 ) | 4.83 ( 0.94 ) | 2.27 ( 0.17 ) |
| Jaw clicking | 2 | 17.33 ( 1.57 - 191.09 ) | 17.33 ( 10.26 ) | 6.44 ( 0.86 ) | 2.69 ( 0.48 ) |
|  |  |  |  |  |  |

Abbreviation: Asterisks (*) indicate statistically significant signals in algorithm; ROR, reporting odds ratio; PRR, proportional reporting ratio; EBGM, empirical Bayesian geometric mean; EBGM05, the lower limit of the 95% CI of EBGM; IC, information component; IC025, the lower limit of the 95% CI of the IC; CI, confidence interval; PT,preferred term; AEs, adverse events.

Supplementary Table S4: Top 50 most frequent adverse events for Ofatumumab at the preferred term (PT) level in males from FAERS data.

| PT | Case numbers | ROR(95%Cl) | PRR(χ^2^) | EBGM  (EBGM05) | IC(IC025) |
| --- | --- | --- | --- | --- | --- |
| Fatigue* | 647 | 2.26 ( 2.07 - 2.47 ) | 2.2 ( 345.85 ) | 1.95 ( 1.82 ) | 0.97 ( 0.84 ) |
| Chills* | 561 | 18.27 ( 15.8 - 21.14 ) | 17.51 ( 2885.43 ) | 6.42 ( 5.68 ) | 2.68 ( 2.52 ) |
| Pyrexia* | 531 | 6.43 ( 5.74 - 7.2 ) | 6.2 ( 1357.38 ) | 4.02 ( 3.65 ) | 2.01 ( 1.86 ) |
| Headache* | 489 | 3.29 ( 2.96 - 3.66 ) | 3.2 ( 546.86 ) | 2.6 ( 2.38 ) | 1.38 ( 1.23 ) |
| Influenza like illness* | 462 | 10.19 ( 8.9 - 11.66 ) | 9.85 ( 1719.75 ) | 5.11 ( 4.57 ) | 2.35 ( 2.19 ) |
| Pain* | 399 | 3.18 ( 2.84 - 3.58 ) | 3.12 ( 425.48 ) | 2.55 ( 2.31 ) | 1.35 ( 1.19 ) |
| Multiple sclerosis relapse | 200 | 0.55 ( 0.48 - 0.64 ) | 0.56 ( 66.59 ) | 0.59 ( 0.52 ) | -0.77 ( -0.98 ) |
| Gait disturbance | 196 | 1.03 ( 0.89 - 1.2 ) | 1.03 ( 0.16 ) | 1.03 ( 0.91 ) | 0.04 ( -0.18 ) |
| Drug ineffective | 192 | 0.99 ( 0.85 - 1.16 ) | 0.99 ( 0.01 ) | 0.99 ( 0.88 ) | -0.01 ( -0.23 ) |
| Asthenia* | 188 | 1.39 ( 1.19 - 1.63 ) | 1.39 ( 17.77 ) | 1.33 ( 1.17 ) | 0.42 ( 0.19 ) |
| Hypoaesthesia* | 188 | 1.46 ( 1.25 - 1.71 ) | 1.46 ( 23.32 ) | 1.39 ( 1.22 ) | 0.48 ( 0.25 ) |
| Covid-19 | 167 | 0.42 ( 0.36 - 0.49 ) | 0.42 ( 128.61 ) | 0.45 ( 0.4 ) | -1.15 ( -1.38 ) |
| Dizziness* | 162 | 1.47 ( 1.24 - 1.73 ) | 1.46 ( 20.23 ) | 1.39 ( 1.21 ) | 0.48 ( 0.24 ) |
| Feeling abnormal* | 157 | 2.42 ( 2.02 - 2.89 ) | 2.4 ( 100.88 ) | 2.09 ( 1.8 ) | 1.07 ( 0.81 ) |
| Nausea* | 147 | 1.77 ( 1.48 - 2.12 ) | 1.76 ( 40.54 ) | 1.63 ( 1.41 ) | 0.71 ( 0.45 ) |
| Multiple sclerosis | 145 | 0.49 ( 0.41 - 0.58 ) | 0.5 ( 71.76 ) | 0.52 ( 0.45 ) | -0.93 ( -1.18 ) |
| Malaise* | 134 | 1.94 ( 1.6 - 2.34 ) | 1.93 ( 48.98 ) | 1.76 ( 1.5 ) | 0.81 ( 0.54 ) |
| Myalgia* | 131 | 5.76 ( 4.62 - 7.2 ) | 5.72 ( 306.32 ) | 3.83 ( 3.18 ) | 1.94 ( 1.64 ) |
| Inappropriate schedule of product administration* | 125 | 3.94 ( 3.19 - 4.88 ) | 3.92 ( 186.76 ) | 3 ( 2.51 ) | 1.58 ( 1.29 ) |
| Muscle spasms* | 121 | 2 ( 1.64 - 2.43 ) | 1.99 ( 48.33 ) | 1.8 ( 1.52 ) | 0.85 ( 0.56 ) |
| Balance disorder | 119 | 1.03 ( 0.85 - 1.24 ) | 1.03 ( 0.08 ) | 1.02 ( 0.87 ) | 0.03 ( -0.24 ) |
| Muscular weakness* | 118 | 1.25 ( 1.03 - 1.52 ) | 1.25 ( 5.15 ) | 1.22 ( 1.03 ) | 0.28 ( 0 ) |
| Fall | 114 | 0.57 ( 0.47 - 0.69 ) | 0.57 ( 34.87 ) | 0.6 ( 0.51 ) | -0.74 ( -1.02 ) |
| Nasopharyngitis* | 110 | 1.85 ( 1.51 - 2.28 ) | 1.84 ( 35.16 ) | 1.69 ( 1.43 ) | 0.76 ( 0.46 ) |
| Tremor* | 107 | 1.88 ( 1.53 - 2.32 ) | 1.88 ( 36.01 ) | 1.72 ( 1.44 ) | 0.78 ( 0.48 ) |
| Arthralgia* | 104 | 1.77 ( 1.44 - 2.19 ) | 1.77 ( 28.9 ) | 1.64 ( 1.37 ) | 0.71 ( 0.4 ) |
| Pain in extremity | 103 | 1.2 ( 0.97 - 1.47 ) | 1.19 ( 2.86 ) | 1.17 ( 0.98 ) | 0.23 ( -0.07 ) |
| Back pain* | 101 | 1.55 ( 1.25 - 1.92 ) | 1.55 ( 16.67 ) | 1.46 ( 1.23 ) | 0.55 ( 0.24 ) |
| Product dose omission issue | 97 | 0.62 ( 0.5 - 0.76 ) | 0.62 ( 20.98 ) | 0.65 ( 0.54 ) | -0.63 ( -0.93 ) |
| Vomiting* | 95 | 1.92 ( 1.54 - 2.4 ) | 1.92 ( 34.14 ) | 1.75 ( 1.45 ) | 0.81 ( 0.48 ) |
| Injection site pain* | 92 | 3.68 ( 2.88 - 4.7 ) | 3.66 ( 124.83 ) | 2.86 ( 2.33 ) | 1.52 ( 1.18 ) |
| Insomnia* | 85 | 1.3 ( 1.03 - 1.63 ) | 1.3 ( 5.01 ) | 1.26 ( 1.04 ) | 0.33 ( 0 ) |
| Cough* | 83 | 1.62 ( 1.28 - 2.06 ) | 1.62 ( 16.65 ) | 1.52 ( 1.25 ) | 0.61 ( 0.27 ) |
| Paraesthesia | 81 | 1.02 ( 0.81 - 1.29 ) | 1.02 ( 0.04 ) | 1.02 ( 0.84 ) | 0.03 ( -0.3 ) |
| Drug effect less than expected* | 76 | 36.32 ( 21.72 - 60.73 ) | 36.11 ( 497.15 ) | 7.72 ( 5.02 ) | 2.95 ( 2.51 ) |
| Accidental exposure to product* | 74 | 79.56 ( 38.35 - 165.05 ) | 79.1 ( 557.11 ) | 8.62 ( 4.68 ) | 3.11 ( 2.65 ) |
| Memory impairment | 70 | 0.56 ( 0.44 - 0.72 ) | 0.56 ( 22.24 ) | 0.59 ( 0.48 ) | -0.76 ( -1.11 ) |
| Mobility decreased | 65 | 0.78 ( 0.6 - 1 ) | 0.78 ( 3.78 ) | 0.8 ( 0.64 ) | -0.33 ( -0.7 ) |
| Hyperhidrosis* | 64 | 3.39 ( 2.54 - 4.53 ) | 3.38 ( 76.96 ) | 2.7 ( 2.12 ) | 1.44 ( 1.03 ) |
| Rhinorrhoea* | 60 | 3.65 ( 2.7 - 4.94 ) | 3.64 ( 80.68 ) | 2.85 ( 2.21 ) | 1.51 ( 1.09 ) |
| Oropharyngeal pain* | 59 | 2.75 ( 2.05 - 3.69 ) | 2.74 ( 49.54 ) | 2.32 ( 1.81 ) | 1.21 ( 0.8 ) |
| Illness* | 56 | 1.45 ( 1.09 - 1.92 ) | 1.45 ( 6.63 ) | 1.38 ( 1.09 ) | 0.47 ( 0.06 ) |
| Somnolence* | 55 | 1.64 ( 1.23 - 2.19 ) | 1.64 ( 11.53 ) | 1.54 ( 1.21 ) | 0.62 ( 0.2 ) |
| Urinary tract infection | 54 | 0.42 ( 0.32 - 0.55 ) | 0.42 ( 42.24 ) | 0.44 ( 0.35 ) | -1.17 ( -1.57 ) |
| Rash | 54 | 1.19 ( 0.9 - 1.58 ) | 1.19 ( 1.45 ) | 1.17 ( 0.92 ) | 0.22 ( -0.19 ) |
| Diarrhoea | 51 | 0.56 ( 0.42 - 0.74 ) | 0.56 ( 16.83 ) | 0.59 ( 0.46 ) | -0.77 ( -1.19 ) |
| Visual impairment | 50 | 0.81 ( 0.6 - 1.08 ) | 0.81 ( 2.09 ) | 0.82 ( 0.65 ) | -0.28 ( -0.7 ) |
| Pruritus | 49 | 0.83 ( 0.62 - 1.12 ) | 0.83 ( 1.47 ) | 0.85 ( 0.66 ) | -0.24 ( -0.66 ) |
| Dyspnoea | 49 | 0.85 ( 0.63 - 1.14 ) | 0.85 ( 1.19 ) | 0.86 ( 0.68 ) | -0.21 ( -0.64 ) |
| Therapeutic response shortened* | 48 | 5.88 ( 4.07 - 8.5 ) | 5.86 ( 114.99 ) | 3.89 ( 2.86 ) | 1.96 ( 1.47 ) |

Abbreviation: Asterisks (*) indicate statistically significant signals in algorithm; ROR, reporting odds ratio; PRR, proportional reporting ratio; EBGM, empirical Bayesian geometric mean; EBGM05, the lower limit of the 95% CI of EBGM; IC, information component; IC025, the lower limit of the 95% CI of the IC; CI, confidence interval; PT,preferred term; AEs, adverse events.

Supplementary Table S5: Top 50 most frequent adverse events for Ofatumumab at the preferred term (PT) level in females from FAERS data.

| PT | Case numbers | ROR(95%Cl) | PRR(χ^2^) | EBGM  (EBGM05) | IC(IC025) |
| --- | --- | --- | --- | --- | --- |
| Fatigue* | 2,586 | 2.06 ( 1.97 - 2.16 ) | 2.01 ( 1088.33 ) | 1.81 ( 1.75 ) | 0.86 ( 0.8 ) |
| Headache* | 2,374 | 2.93 ( 2.79 - 3.07 ) | 2.84 ( 2158.97 ) | 2.38 ( 2.28 ) | 1.25 ( 1.18 ) |
| Pain* | 1,723 | 3.2 ( 3.03 - 3.38 ) | 3.12 ( 1842.01 ) | 2.55 ( 2.43 ) | 1.35 ( 1.27 ) |
| Chills* | 1,619 | 12.98 ( 12.01 - 14.02 ) | 12.58 ( 6994.56 ) | 5.67 ( 5.31 ) | 2.5 ( 2.41 ) |
| Pyrexia* | 1,547 | 5.95 ( 5.57 - 6.35 ) | 5.79 ( 3674.71 ) | 3.85 ( 3.64 ) | 1.94 ( 1.86 ) |
| Influenza like illness* | 1,407 | 7.86 ( 7.31 - 8.45 ) | 7.66 ( 4312.4 ) | 4.5 ( 4.24 ) | 2.17 ( 2.08 ) |
| Nausea* | 910 | 1.6 ( 1.49 - 1.72 ) | 1.59 ( 169.28 ) | 1.5 ( 1.41 ) | 0.58 ( 0.48 ) |
| Covid-19 | 706 | 0.62 ( 0.58 - 0.67 ) | 0.63 ( 146.94 ) | 0.65 ( 0.61 ) | -0.61 ( -0.72 ) |
| Multiple sclerosis relapse | 699 | 0.52 ( 0.49 - 0.57 ) | 0.53 ( 279.69 ) | 0.56 ( 0.52 ) | -0.84 ( -0.95 ) |
| Asthenia* | 616 | 1.36 ( 1.25 - 1.48 ) | 1.36 ( 50.49 ) | 1.31 ( 1.22 ) | 0.39 ( 0.26 ) |
| Hypoaesthesia* | 603 | 1.28 ( 1.17 - 1.39 ) | 1.27 ( 31.08 ) | 1.24 ( 1.15 ) | 0.31 ( 0.18 ) |
| Dizziness* | 557 | 1.14 ( 1.05 - 1.25 ) | 1.14 ( 8.71 ) | 1.12 ( 1.04 ) | 0.17 ( 0.04 ) |
| Pain in extremity* | 557 | 1.46 ( 1.33 - 1.59 ) | 1.45 ( 67.4 ) | 1.39 ( 1.28 ) | 0.47 ( 0.34 ) |
| Feeling abnormal* | 549 | 1.97 ( 1.79 - 2.16 ) | 1.96 ( 209.62 ) | 1.78 ( 1.64 ) | 0.83 ( 0.69 ) |
| Gait disturbance | 544 | 0.98 ( 0.9 - 1.08 ) | 0.98 ( 0.14 ) | 0.99 ( 0.91 ) | -0.02 ( -0.15 ) |
| Malaise* | 526 | 1.65 ( 1.5 - 1.81 ) | 1.64 ( 110.45 ) | 1.54 ( 1.42 ) | 0.62 ( 0.48 ) |
| Nasopharyngitis* | 516 | 1.88 ( 1.71 - 2.07 ) | 1.87 ( 172.45 ) | 1.71 ( 1.58 ) | 0.78 ( 0.64 ) |
| Injection site pain* | 490 | 3.49 ( 3.14 - 3.88 ) | 3.46 ( 612.28 ) | 2.75 ( 2.52 ) | 1.46 ( 1.31 ) |
| Drug ineffective | 471 | 0.8 ( 0.73 - 0.88 ) | 0.8 ( 20.84 ) | 0.82 ( 0.76 ) | -0.28 ( -0.42 ) |
| Arthralgia* | 436 | 1.8 ( 1.62 - 1.99 ) | 1.79 ( 126.07 ) | 1.65 ( 1.51 ) | 0.72 ( 0.57 ) |
| Inappropriate schedule of product administration* | 431 | 3.5 ( 3.13 - 3.91 ) | 3.48 ( 540.95 ) | 2.76 ( 2.51 ) | 1.46 ( 1.3 ) |
| Back pain* | 420 | 1.62 ( 1.46 - 1.8 ) | 1.61 ( 82.68 ) | 1.52 ( 1.39 ) | 0.6 ( 0.45 ) |
| Myalgia* | 403 | 4.66 ( 4.13 - 5.26 ) | 4.63 ( 744.13 ) | 3.35 ( 3.02 ) | 1.74 ( 1.58 ) |
| Cough* | 394 | 1.67 ( 1.5 - 1.86 ) | 1.66 ( 87.32 ) | 1.55 ( 1.42 ) | 0.64 ( 0.48 ) |
| Fall | 380 | 0.54 ( 0.49 - 0.6 ) | 0.55 ( 137.26 ) | 0.57 ( 0.52 ) | -0.8 ( -0.96 ) |
| Urinary tract infection | 375 | 0.79 ( 0.71 - 0.88 ) | 0.79 ( 18.78 ) | 0.81 ( 0.74 ) | -0.3 ( -0.46 ) |
| Vomiting* | 374 | 1.28 ( 1.14 - 1.42 ) | 1.27 ( 19.26 ) | 1.24 ( 1.13 ) | 0.31 ( 0.15 ) |
| Multiple sclerosis | 354 | 0.39 ( 0.35 - 0.43 ) | 0.39 ( 326.26 ) | 0.42 ( 0.38 ) | -1.26 ( -1.42 ) |
| Muscular weakness* | 349 | 1.18 ( 1.05 - 1.32 ) | 1.17 ( 8 ) | 1.15 ( 1.05 ) | 0.21 ( 0.04 ) |
| Paraesthesia | 349 | 1.03 ( 0.92 - 1.15 ) | 1.03 ( 0.28 ) | 1.03 ( 0.94 ) | 0.04 ( -0.12 ) |
| Muscle spasms* | 347 | 1.44 ( 1.28 - 1.61 ) | 1.43 ( 38.95 ) | 1.37 ( 1.25 ) | 0.45 ( 0.29 ) |
| Tremor* | 329 | 1.68 ( 1.5 - 1.9 ) | 1.68 ( 75.72 ) | 1.57 ( 1.42 ) | 0.65 ( 0.48 ) |
| Product dose omission issue | 319 | 0.55 ( 0.49 - 0.61 ) | 0.55 ( 111.44 ) | 0.58 ( 0.53 ) | -0.79 ( -0.96 ) |
| Accidental exposure to product* | 306 | 90.16 ( 61.6 - 131.96 ) | 89.59 ( 2322.3 ) | 8.67 ( 6.3 ) | 3.12 ( 2.89 ) |
| Balance disorder | 300 | 0.88 ( 0.78 - 0.99 ) | 0.88 ( 4.2 ) | 0.89 ( 0.81 ) | -0.16 ( -0.34 ) |
| Illness* | 294 | 1.33 ( 1.17 - 1.5 ) | 1.32 ( 20.22 ) | 1.28 ( 1.15 ) | 0.36 ( 0.18 ) |
| Injection site bruising* | 293 | 5.11 ( 4.42 - 5.91 ) | 5.09 ( 602.74 ) | 3.56 ( 3.15 ) | 1.83 ( 1.63 ) |
| Oropharyngeal pain* | 290 | 2.44 ( 2.14 - 2.78 ) | 2.43 ( 190 ) | 2.11 ( 1.89 ) | 1.08 ( 0.89 ) |
| Migraine* | 288 | 1.76 ( 1.55 - 2 ) | 1.76 ( 78.27 ) | 1.63 ( 1.46 ) | 0.7 ( 0.52 ) |
| Memory impairment | 281 | 0.6 ( 0.53 - 0.68 ) | 0.6 ( 69.95 ) | 0.63 ( 0.57 ) | -0.67 ( -0.85 ) |
| Insomnia* | 247 | 1.16 ( 1.01 - 1.32 ) | 1.16 ( 4.68 ) | 1.14 ( 1.02 ) | 0.19 ( -0.01 ) |
| Diarrhoea | 241 | 0.54 ( 0.47 - 0.61 ) | 0.54 ( 90.67 ) | 0.57 ( 0.51 ) | -0.82 ( -1.01 ) |
| Pruritus | 230 | 0.75 ( 0.65 - 0.86 ) | 0.75 ( 18.02 ) | 0.77 ( 0.69 ) | -0.38 ( -0.58 ) |
| Rhinorrhoea* | 227 | 3.09 ( 2.66 - 3.6 ) | 3.08 ( 234.92 ) | 2.53 ( 2.23 ) | 1.34 ( 1.12 ) |
| Drug effect less than expected* | 219 | 32.2 ( 24.1 - 43.02 ) | 32.06 ( 1380.7 ) | 7.5 ( 5.89 ) | 2.91 ( 2.65 ) |
| Rash | 217 | 1.05 ( 0.91 - 1.2 ) | 1.05 ( 0.39 ) | 1.04 ( 0.92 ) | 0.06 ( -0.15 ) |
| Dyspnoea | 212 | 0.73 ( 0.64 - 0.84 ) | 0.74 ( 18.75 ) | 0.76 ( 0.67 ) | -0.4 ( -0.61 ) |
| Stress | 211 | 1 ( 0.86 - 1.15 ) | 1 ( 0 ) | 1 ( 0.88 ) | 0 ( -0.21 ) |
| Alopecia | 209 | 0.65 ( 0.57 - 0.75 ) | 0.65 ( 36.17 ) | 0.68 ( 0.6 ) | -0.56 ( -0.77 ) |
| Injection site haemorrhage* | 200 | 6 ( 5.01 - 7.19 ) | 5.98 ( 487.16 ) | 3.92 ( 3.37 ) | 1.97 ( 1.73 ) |

Abbreviation: Asterisks (*) indicate statistically significant signals in algorithm; ROR, reporting odds ratio; PRR, proportional reporting ratio; EBGM, empirical Bayesian geometric mean; EBGM05, the lower limit of the 95% CI of EBGM; IC, information component; IC025, the lower limit of the 95% CI of the IC; CI, confidence interval; PT,preferred term; AEs, adverse events.

Supplementary Table S6: Adverse events at the PT level for Ofatumumab in patients aged under 18 from FAERS data.

| PT | Case numbers | ROR(95%Cl) | PRR(χ^2^) | EBGM  (EBGM05) | IC(IC025) |
| --- | --- | --- | --- | --- | --- |
| Chills* | 4 | 56.45 ( 14.57 - 218.7 ) | 51.04 ( 109.4 ) | 28.8 ( 9.27 ) | 4.85 ( 3.21 ) |
| Nausea* | 3 | 8.53 ( 2.46 - 29.53 ) | 7.98 ( 16.44 ) | 7.2 ( 2.55 ) | 2.85 ( 1.25 ) |
| Pyrexia* | 2 | 4.57 ( 1.05 - 19.84 ) | 4.4 ( 4.97 ) | 4.18 ( 1.22 ) | 2.06 ( 0.27 ) |
| Dizziness* | 2 | 3.78 ( 0.88 - 16.28 ) | 3.65 ( 3.68 ) | 3.5 ( 1.03 ) | 1.81 ( 0.02 ) |
| Influenza like illness* | 2 | 12.14 ( 2.6 - 56.62 ) | 11.6 ( 16.47 ) | 9.97 ( 2.75 ) | 3.32 ( 1.43 ) |
| Pain* | 2 | 7.84 ( 1.75 - 35.1 ) | 7.51 ( 10.16 ) | 6.82 ( 1.95 ) | 2.77 ( 0.93 ) |
| Vomiting* | 2 | 5.78 ( 1.32 - 25.38 ) | 5.55 ( 6.93 ) | 5.18 ( 1.5 ) | 2.37 ( 0.56 ) |
| Off label use | 2 | 0.63 ( 0.15 - 2.64 ) | 0.65 ( 0.4 ) | 0.65 ( 0.2 ) | -0.61 ( -2.35 ) |
| Back pain | 1 | 4.06 ( 0.53 - 31.38 ) | 3.99 ( 2.12 ) | 3.81 ( 0.69 ) | 1.93 ( -0.26 ) |
| Myalgia* | 1 | 21.77 ( 2.22 - 213.88 ) | 21.27 ( 14.51 ) | 16.2 ( 2.4 ) | 4.02 ( 1.57 ) |
| Injection related reaction* | 1 | Inf ( NaN - Inf ) | Inf ( 63.83 ) | 64.8 ( 0 ) | 6.02 ( 3.1 ) |
| Multiple sclerosis relapse | 1 | 0.64 ( 0.09 - 4.72 ) | 0.65 ( 0.19 ) | 0.65 ( 0.12 ) | -0.61 ( -2.72 ) |
| Adjustment disorder with depressed mood* | 1 | Inf ( NaN - Inf ) | Inf ( 63.83 ) | 64.8 ( 0 ) | 6.02 ( 3.1 ) |
| Paraesthesia | 1 | 3.42 ( 0.45 - 26.15 ) | 3.36 ( 1.59 ) | 3.24 ( 0.59 ) | 1.7 ( -0.48 ) |
| Accidental exposure to product by child* | 1 | Inf ( NaN - Inf ) | Inf ( 63.83 ) | 64.8 ( 0 ) | 6.02 ( 3.1 ) |
| Malaise | 1 | 3.61 ( 0.47 - 27.69 ) | 3.54 ( 1.74 ) | 3.41 ( 0.62 ) | 1.77 ( -0.41 ) |
| Diarrhoea | 1 | 5.01 ( 0.64 - 39.19 ) | 4.91 ( 2.9 ) | 4.63 ( 0.83 ) | 2.21 ( 0 ) |
| Body temperature abnormal* | 1 | Inf ( NaN - Inf ) | Inf ( 63.83 ) | 64.8 ( 0 ) | 6.02 ( 3.1 ) |
| Influenza* | 1 | 6.52 ( 0.81 - 52.11 ) | 6.38 ( 4.14 ) | 5.89 ( 1.03 ) | 2.56 ( 0.31 ) |
| Abdominal pain upper | 1 | 2.82 ( 0.37 - 21.38 ) | 2.77 ( 1.1 ) | 2.7 ( 0.5 ) | 1.43 ( -0.73 ) |
| Dizziness postural* | 1 | 21.77 ( 2.22 - 213.88 ) | 21.27 ( 14.51 ) | 16.2 ( 2.4 ) | 4.02 ( 1.57 ) |
| Gastroenteritis viral* | 1 | 65.38 ( 4.02 - 1063.72 ) | 63.8 ( 30.93 ) | 32.4 ( 3.14 ) | 5.02 ( 2.35 ) |
| Heart rate increased* | 1 | 7.24 ( 0.9 - 58.52 ) | 7.09 ( 4.73 ) | 6.48 ( 1.13 ) | 2.7 ( 0.44 ) |
| Somnolence | 1 | 2.82 ( 0.37 - 21.38 ) | 2.77 ( 1.1 ) | 2.7 ( 0.5 ) | 1.43 ( -0.73 ) |
| Hypotension* | 1 | 7.24 ( 0.9 - 58.52 ) | 7.09 ( 4.73 ) | 6.48 ( 1.13 ) | 2.7 ( 0.44 ) |
| Aspartate aminotransferase increased* | 1 | Inf ( NaN - Inf ) | Inf ( 63.83 ) | 64.8 ( 0 ) | 6.02 ( 3.1 ) |
| Blood creatine phosphokinase increased* | 1 | 32.67 ( 2.9 - 367.72 ) | 31.9 ( 19.98 ) | 21.6 ( 2.85 ) | 4.43 ( 1.9 ) |
| Alanine aminotransferase increased* | 1 | Inf ( NaN - Inf ) | Inf ( 63.83 ) | 64.8 ( 0 ) | 6.02 ( 3.1 ) |
| Liver function test increased* | 1 | 65.38 ( 4.02 - 1063.72 ) | 63.8 ( 30.93 ) | 32.4 ( 3.14 ) | 5.02 ( 2.35 ) |
| Product use issue* | 1 | 6.52 ( 0.81 - 52.11 ) | 6.38 ( 4.14 ) | 5.89 ( 1.03 ) | 2.56 ( 0.31 ) |

Abbreviation: Asterisks (*) indicate statistically significant signals in algorithm; ROR, reporting odds ratio; PRR, proportional reporting ratio; EBGM, empirical Bayesian geometric mean; EBGM05, the lower limit of the 95% CI of EBGM; IC, information component; IC025, the lower limit of the 95% CI of the IC; CI, confidence interval; PT,preferred term; AEs, adverse events.

Supplementary Table S7: Adverse events at the PT level for Ofatumumab in patients aged 18 to under 45 from FAERS data.

| PT | Case numbers | ROR(95%Cl) | PRR(χ^2^) | EBGM  (EBGM05) | IC(IC025) |
| --- | --- | --- | --- | --- | --- |
| Fatigue* | 956 | 1.99 ( 1.85 - 2.14 ) | 1.94 ( 355.74 ) | 1.75 ( 1.64 ) | 0.8 ( 0.7 ) |
| Headache* | 844 | 2.39 ( 2.21 - 2.59 ) | 2.33 ( 501.52 ) | 2.02 ( 1.89 ) | 1.01 ( 0.9 ) |
| Pyrexia* | 698 | 4.93 ( 4.48 - 5.43 ) | 4.78 ( 1300.38 ) | 3.33 ( 3.07 ) | 1.73 ( 1.61 ) |
| Chills* | 654 | 11.28 ( 10 - 12.72 ) | 10.9 ( 2446.72 ) | 5.09 ( 4.6 ) | 2.35 ( 2.2 ) |
| Pain* | 641 | 3.43 ( 3.12 - 3.77 ) | 3.34 ( 743.55 ) | 2.63 ( 2.43 ) | 1.4 ( 1.27 ) |
| Influenza like illness* | 542 | 8.59 ( 7.6 - 9.71 ) | 8.36 ( 1692.13 ) | 4.52 ( 4.08 ) | 2.18 ( 2.02 ) |
| Nausea* | 310 | 1.37 ( 1.21 - 1.55 ) | 1.36 ( 25.72 ) | 1.31 ( 1.18 ) | 0.39 ( 0.21 ) |
| Hypoaesthesia* | 272 | 1.35 ( 1.18 - 1.53 ) | 1.34 ( 20.28 ) | 1.29 ( 1.16 ) | 0.37 ( 0.18 ) |
| Covid-19 | 246 | 0.48 ( 0.42 - 0.55 ) | 0.49 ( 126.22 ) | 0.52 ( 0.47 ) | -0.94 ( -1.13 ) |
| Nasopharyngitis* | 223 | 1.58 ( 1.37 - 1.83 ) | 1.57 ( 38.84 ) | 1.47 ( 1.31 ) | 0.56 ( 0.35 ) |
| Multiple sclerosis relapse | 220 | 0.44 ( 0.38 - 0.5 ) | 0.44 ( 147.99 ) | 0.48 ( 0.42 ) | -1.07 ( -1.27 ) |
| Dizziness | 210 | 1.03 ( 0.89 - 1.19 ) | 1.03 ( 0.13 ) | 1.02 ( 0.91 ) | 0.03 ( -0.18 ) |
| Asthenia* | 210 | 1.34 ( 1.16 - 1.56 ) | 1.34 ( 15.55 ) | 1.29 ( 1.14 ) | 0.37 ( 0.15 ) |
| Injection site pain* | 206 | 4.72 ( 3.96 - 5.61 ) | 4.67 ( 370.95 ) | 3.28 ( 2.84 ) | 1.71 ( 1.48 ) |
| Malaise* | 194 | 1.85 ( 1.58 - 2.16 ) | 1.84 ( 60.06 ) | 1.68 ( 1.47 ) | 0.74 ( 0.52 ) |
| Feeling abnormal* | 191 | 1.98 ( 1.69 - 2.33 ) | 1.97 ( 73.18 ) | 1.77 ( 1.55 ) | 0.83 ( 0.6 ) |
| Pain in extremity | 170 | 1.14 ( 0.97 - 1.35 ) | 1.14 ( 2.66 ) | 1.12 ( 0.98 ) | 0.17 ( -0.07 ) |
| Myalgia* | 168 | 4.17 ( 3.46 - 5.04 ) | 4.14 ( 260.87 ) | 3.04 ( 2.6 ) | 1.6 ( 1.35 ) |
| Inappropriate schedule of product administration* | 167 | 4.01 ( 3.32 - 4.83 ) | 3.98 ( 245.88 ) | 2.96 ( 2.53 ) | 1.57 ( 1.31 ) |
| Vomiting* | 166 | 1.43 ( 1.21 - 1.69 ) | 1.43 ( 17.91 ) | 1.36 ( 1.18 ) | 0.44 ( 0.2 ) |
| Back pain* | 156 | 1.5 ( 1.26 - 1.78 ) | 1.49 ( 21.43 ) | 1.41 ( 1.22 ) | 0.5 ( 0.25 ) |
| Gait disturbance | 156 | 1.03 ( 0.87 - 1.22 ) | 1.03 ( 0.1 ) | 1.02 ( 0.89 ) | 0.03 ( -0.21 ) |
| Arthralgia* | 149 | 1.76 ( 1.47 - 2.1 ) | 1.75 ( 39.19 ) | 1.61 ( 1.39 ) | 0.69 ( 0.43 ) |
| Paraesthesia | 149 | 1.13 ( 0.95 - 1.35 ) | 1.13 ( 2.03 ) | 1.12 ( 0.97 ) | 0.16 ( -0.09 ) |
| Cough* | 148 | 1.33 ( 1.12 - 1.58 ) | 1.33 ( 10.26 ) | 1.28 ( 1.1 ) | 0.36 ( 0.1 ) |
| Tremor* | 135 | 1.76 ( 1.46 - 2.12 ) | 1.75 ( 35.63 ) | 1.61 ( 1.38 ) | 0.69 ( 0.42 ) |
| Migraine* | 125 | 1.93 ( 1.58 - 2.35 ) | 1.92 ( 44.33 ) | 1.74 ( 1.47 ) | 0.8 ( 0.51 ) |
| Muscle spasms* | 120 | 1.5 ( 1.23 - 1.83 ) | 1.5 ( 16.75 ) | 1.42 ( 1.2 ) | 0.5 ( 0.22 ) |
| Oropharyngeal pain* | 120 | 1.71 ( 1.4 - 2.08 ) | 1.7 ( 28.5 ) | 1.57 ( 1.33 ) | 0.65 ( 0.37 ) |
| Multiple sclerosis | 115 | 0.46 ( 0.38 - 0.56 ) | 0.46 ( 68.34 ) | 0.49 ( 0.42 ) | -1.02 ( -1.29 ) |
| Muscular weakness | 111 | 1.08 ( 0.88 - 1.31 ) | 1.08 ( 0.53 ) | 1.07 ( 0.9 ) | 0.09 ( -0.2 ) |
| Accidental exposure to product* | 106 | 136.29 ( 59.87 - 310.26 ) | 135.49 ( 758.62 ) | 8.2 ( 4.12 ) | 3.04 ( 2.65 ) |
| Injection site bruising* | 105 | 6.8 ( 5.23 - 8.85 ) | 6.77 ( 274.55 ) | 4.06 ( 3.26 ) | 2.02 ( 1.68 ) |
| Product dose omission issue | 102 | 0.68 ( 0.56 - 0.83 ) | 0.68 ( 13.87 ) | 0.71 ( 0.6 ) | -0.5 ( -0.79 ) |
| Illness* | 101 | 1.98 ( 1.59 - 2.46 ) | 1.97 ( 38.56 ) | 1.77 ( 1.48 ) | 0.83 ( 0.51 ) |
| Insomnia | 99 | 1.01 ( 0.82 - 1.25 ) | 1.01 ( 0.02 ) | 1.01 ( 0.85 ) | 0.02 ( -0.29 ) |
| Pruritus | 99 | 0.85 ( 0.69 - 1.04 ) | 0.85 ( 2.47 ) | 0.86 ( 0.72 ) | -0.21 ( -0.52 ) |
| Urinary tract infection | 97 | 0.78 ( 0.64 - 0.97 ) | 0.78 ( 5.23 ) | 0.8 ( 0.68 ) | -0.31 ( -0.62 ) |
| Balance disorder | 97 | 1.11 ( 0.9 - 1.38 ) | 1.11 ( 0.96 ) | 1.1 ( 0.92 ) | 0.13 ( -0.18 ) |
| Dyspnoea | 89 | 0.66 ( 0.53 - 0.81 ) | 0.66 ( 14.72 ) | 0.68 ( 0.57 ) | -0.55 ( -0.86 ) |
| Drug ineffective | 89 | 0.6 ( 0.48 - 0.74 ) | 0.6 ( 22.46 ) | 0.63 ( 0.52 ) | -0.67 ( -0.99 ) |
| Vision blurred | 88 | 1.06 ( 0.85 - 1.32 ) | 1.06 ( 0.25 ) | 1.05 ( 0.87 ) | 0.07 ( -0.25 ) |
| Rash | 85 | 1 ( 0.8 - 1.26 ) | 1 ( 0 ) | 1 ( 0.83 ) | 0.01 ( -0.32 ) |
| Influenza | 82 | 0.86 ( 0.69 - 1.09 ) | 0.86 ( 1.59 ) | 0.88 ( 0.72 ) | -0.19 ( -0.52 ) |
| Fall | 80 | 0.6 ( 0.48 - 0.75 ) | 0.6 ( 19.96 ) | 0.63 ( 0.52 ) | -0.67 ( -1 ) |
| Rhinorrhoea* | 79 | 1.96 ( 1.53 - 2.51 ) | 1.95 ( 29.42 ) | 1.76 ( 1.43 ) | 0.82 ( 0.46 ) |
| Anxiety | 79 | 0.74 ( 0.59 - 0.94 ) | 0.74 ( 6.42 ) | 0.77 ( 0.63 ) | -0.38 ( -0.72 ) |
| Injection site haemorrhage* | 79 | 9.22 ( 6.64 - 12.78 ) | 9.18 ( 262.34 ) | 4.72 ( 3.59 ) | 2.24 ( 1.84 ) |
| Stress | 77 | 1.05 ( 0.83 - 1.34 ) | 1.05 ( 0.18 ) | 1.05 ( 0.86 ) | 0.07 ( -0.28 ) |
| Diarrhoea | 74 | 0.58 ( 0.45 - 0.73 ) | 0.58 ( 21.44 ) | 0.61 ( 0.5 ) | -0.72 ( -1.07 ) |

Abbreviation: Asterisks (*) indicate statistically significant signals in algorithm; ROR, reporting odds ratio; PRR, proportional reporting ratio; EBGM, empirical Bayesian geometric mean; EBGM05, the lower limit of the 95% CI of EBGM; IC, information component; IC025, the lower limit of the 95% CI of the IC; CI, confidence interval; PT,preferred term; AEs, adverse events.

Supplementary Table S8: Adverse events at the PT level for Ofatumumab in patients aged 45 to under 65 from FAERS data.

| PT | Case numbers | ROR(95%Cl) | PRR(χ^2^) | EBGM  (EBGM05) | IC(IC025) |
| --- | --- | --- | --- | --- | --- |
| Fatigue* | 885 | 1.95 ( 1.81 - 2.1 ) | 1.9 ( 327.62 ) | 1.76 ( 1.65 ) | 0.81 ( 0.71 ) |
| Headache* | 769 | 2.84 ( 2.61 - 3.08 ) | 2.75 ( 684.13 ) | 2.37 ( 2.21 ) | 1.25 ( 1.13 ) |
| Chills* | 611 | 13 ( 11.53 - 14.67 ) | 12.58 ( 2878.36 ) | 6.09 ( 5.5 ) | 2.61 ( 2.46 ) |
| Pain* | 589 | 3.02 ( 2.75 - 3.31 ) | 2.95 ( 591.84 ) | 2.5 ( 2.31 ) | 1.32 ( 1.19 ) |
| Pyrexia* | 532 | 5.41 ( 4.87 - 6.02 ) | 5.28 ( 1211.32 ) | 3.79 ( 3.46 ) | 1.92 ( 1.77 ) |
| Influenza like illness* | 475 | 8.18 ( 7.24 - 9.24 ) | 7.98 ( 1611.89 ) | 4.86 ( 4.39 ) | 2.28 ( 2.12 ) |
| Nausea* | 288 | 1.64 ( 1.45 - 1.86 ) | 1.63 ( 61.3 ) | 1.54 ( 1.39 ) | 0.63 ( 0.44 ) |
| Covid-19 | 276 | 0.59 ( 0.52 - 0.66 ) | 0.59 ( 74.77 ) | 0.62 ( 0.56 ) | -0.7 ( -0.88 ) |
| Asthenia* | 252 | 1.36 ( 1.19 - 1.55 ) | 1.35 ( 20.68 ) | 1.31 ( 1.17 ) | 0.39 ( 0.2 ) |
| Gait disturbance | 251 | 1.11 ( 0.98 - 1.27 ) | 1.11 ( 2.51 ) | 1.1 ( 0.98 ) | 0.14 ( -0.06 ) |
| Pain in extremity* | 228 | 1.59 ( 1.38 - 1.83 ) | 1.58 ( 42.33 ) | 1.5 ( 1.33 ) | 0.59 ( 0.38 ) |
| Multiple sclerosis relapse | 227 | 0.58 ( 0.5 - 0.66 ) | 0.58 ( 65.39 ) | 0.61 ( 0.54 ) | -0.72 ( -0.92 ) |
| Hypoaesthesia* | 213 | 1.28 ( 1.11 - 1.48 ) | 1.28 ( 11.37 ) | 1.24 ( 1.1 ) | 0.32 ( 0.11 ) |
| Feeling abnormal* | 208 | 2.17 ( 1.86 - 2.52 ) | 2.15 ( 106.25 ) | 1.95 ( 1.72 ) | 0.96 ( 0.74 ) |
| Dizziness | 199 | 1.15 ( 1 - 1.34 ) | 1.15 ( 3.65 ) | 1.14 ( 1 ) | 0.19 ( -0.03 ) |
| Arthralgia* | 185 | 1.76 ( 1.5 - 2.06 ) | 1.75 ( 50.73 ) | 1.64 ( 1.43 ) | 0.71 ( 0.48 ) |
| Fall | 179 | 0.58 ( 0.5 - 0.67 ) | 0.58 ( 51.45 ) | 0.61 ( 0.53 ) | -0.72 ( -0.94 ) |
| Muscular weakness* | 169 | 1.37 ( 1.17 - 1.61 ) | 1.37 ( 14.81 ) | 1.32 ( 1.16 ) | 0.4 ( 0.17 ) |
| Nasopharyngitis* | 168 | 1.8 ( 1.52 - 2.12 ) | 1.79 ( 49.95 ) | 1.67 ( 1.45 ) | 0.74 ( 0.5 ) |
| Malaise* | 164 | 1.62 ( 1.38 - 1.92 ) | 1.62 ( 33.53 ) | 1.53 ( 1.33 ) | 0.61 ( 0.37 ) |
| Muscle spasms* | 164 | 1.74 ( 1.47 - 2.06 ) | 1.74 ( 43.69 ) | 1.63 ( 1.41 ) | 0.7 ( 0.46 ) |
| Urinary tract infection | 162 | 0.86 ( 0.73 - 1.01 ) | 0.86 ( 3.42 ) | 0.87 ( 0.76 ) | -0.2 ( -0.43 ) |
| Back pain* | 162 | 1.69 ( 1.43 - 2 ) | 1.69 ( 38.92 ) | 1.59 ( 1.38 ) | 0.67 ( 0.42 ) |
| Myalgia* | 154 | 4.58 ( 3.78 - 5.55 ) | 4.55 ( 292.35 ) | 3.43 ( 2.92 ) | 1.78 ( 1.51 ) |
| Cough* | 150 | 1.7 ( 1.43 - 2.02 ) | 1.69 ( 36.62 ) | 1.59 ( 1.38 ) | 0.67 ( 0.42 ) |
| Inappropriate schedule of product administration* | 147 | 3.92 ( 3.24 - 4.75 ) | 3.9 ( 227.5 ) | 3.08 ( 2.62 ) | 1.62 ( 1.35 ) |
| Injection site pain* | 145 | 3.77 ( 3.11 - 4.56 ) | 3.74 ( 211.86 ) | 2.99 ( 2.55 ) | 1.58 ( 1.31 ) |
| Balance disorder | 128 | 0.93 ( 0.78 - 1.12 ) | 0.93 ( 0.59 ) | 0.94 ( 0.81 ) | -0.09 ( -0.36 ) |
| Vomiting* | 121 | 1.37 ( 1.13 - 1.65 ) | 1.36 ( 10.36 ) | 1.32 ( 1.12 ) | 0.4 ( 0.12 ) |
| Tremor* | 121 | 1.78 ( 1.46 - 2.16 ) | 1.77 ( 34.53 ) | 1.65 ( 1.41 ) | 0.73 ( 0.44 ) |
| Multiple sclerosis | 121 | 0.47 ( 0.39 - 0.57 ) | 0.48 ( 67.57 ) | 0.5 ( 0.43 ) | -1 ( -1.27 ) |
| Paraesthesia | 120 | 0.99 ( 0.82 - 1.19 ) | 0.99 ( 0.02 ) | 0.99 ( 0.84 ) | -0.02 ( -0.29 ) |
| Drug ineffective | 118 | 0.8 ( 0.66 - 0.97 ) | 0.8 ( 5.35 ) | 0.82 ( 0.7 ) | -0.29 ( -0.57 ) |
| Insomnia | 106 | 1.19 ( 0.97 - 1.46 ) | 1.19 ( 2.9 ) | 1.17 ( 0.99 ) | 0.23 ( -0.07 ) |
| Memory impairment | 105 | 0.78 ( 0.64 - 0.95 ) | 0.78 ( 6.15 ) | 0.8 ( 0.67 ) | -0.33 ( -0.62 ) |
| Accidental exposure to product* | 105 | 86.8 ( 47.76 - 157.77 ) | 86.28 ( 908.32 ) | 9.75 ( 5.91 ) | 3.28 ( 2.9 ) |
| Injection site bruising* | 103 | 7.19 ( 5.58 - 9.27 ) | 7.15 ( 316.35 ) | 4.57 ( 3.69 ) | 2.19 ( 1.85 ) |
| Diarrhoea | 99 | 0.62 ( 0.5 - 0.76 ) | 0.62 ( 22.04 ) | 0.64 ( 0.54 ) | -0.64 ( -0.94 ) |
| Product dose omission issue | 95 | 0.51 ( 0.42 - 0.63 ) | 0.51 ( 41.89 ) | 0.54 ( 0.45 ) | -0.89 ( -1.2 ) |
| Oropharyngeal pain* | 89 | 2.21 ( 1.75 - 2.78 ) | 2.2 ( 47.72 ) | 1.98 ( 1.63 ) | 0.99 ( 0.65 ) |
| Limb discomfort* | 87 | 2.1 ( 1.66 - 2.65 ) | 2.09 ( 41.04 ) | 1.9 ( 1.57 ) | 0.93 ( 0.59 ) |
| Rhinorrhoea* | 83 | 3.59 ( 2.79 - 4.61 ) | 3.57 ( 113.12 ) | 2.89 ( 2.34 ) | 1.53 ( 1.18 ) |
| Musculoskeletal stiffness* | 80 | 1.32 ( 1.05 - 1.67 ) | 1.32 ( 5.47 ) | 1.28 ( 1.05 ) | 0.36 ( 0.02 ) |
| Mobility decreased | 79 | 0.85 ( 0.68 - 1.07 ) | 0.85 ( 1.85 ) | 0.86 ( 0.71 ) | -0.21 ( -0.55 ) |
| Rash | 76 | 1.14 ( 0.9 - 1.45 ) | 1.14 ( 1.16 ) | 1.12 ( 0.92 ) | 0.17 ( -0.18 ) |
| Stress | 76 | 1.18 ( 0.93 - 1.5 ) | 1.18 ( 1.91 ) | 1.16 ( 0.95 ) | 0.22 ( -0.13 ) |
| Pruritus | 73 | 0.75 ( 0.59 - 0.95 ) | 0.75 ( 5.82 ) | 0.77 ( 0.63 ) | -0.39 ( -0.73 ) |
| Migraine* | 69 | 1.39 ( 1.08 - 1.79 ) | 1.39 ( 6.6 ) | 1.34 ( 1.09 ) | 0.42 ( 0.06 ) |
| Dyspnoea | 68 | 0.68 ( 0.53 - 0.87 ) | 0.68 ( 9.68 ) | 0.7 ( 0.57 ) | -0.51 ( -0.87 ) |
| Illness | 67 | 1.16 ( 0.9 - 1.49 ) | 1.16 ( 1.31 ) | 1.14 ( 0.92 ) | 0.19 ( -0.18 ) |

Abbreviation: Asterisks (*) indicate statistically significant signals in algorithm; ROR, reporting odds ratio; PRR, proportional reporting ratio; EBGM, empirical Bayesian geometric mean; EBGM05, the lower limit of the 95% CI of EBGM; IC, information component; IC025, the lower limit of the 95% CI of the IC; CI, confidence interval; PT,preferred term; AEs, adverse events.

Supplementary Table S9: Adverse events at the PT level for Ofatumumab in patients aged 65 and above from FAERS data.

| PT | Case numbers | ROR(95%Cl) | PRR(χ^2^) | EBGM  (EBGM05) | IC(IC025) |
| --- | --- | --- | --- | --- | --- |
| Headache* | 68 | 3.65 ( 2.81 - 4.74 ) | 3.54 ( 107.35 ) | 3.17 ( 2.55 ) | 1.67 ( 1.29 ) |
| Fatigue* | 61 | 1.75 ( 1.34 - 2.29 ) | 1.73 ( 17.64 ) | 1.67 ( 1.34 ) | 0.74 ( 0.36 ) |
| Chills* | 59 | 13.71 ( 9.86 - 19.08 ) | 13.28 ( 411.47 ) | 8.51 ( 6.46 ) | 3.09 ( 2.65 ) |
| Pain* | 44 | 2.31 ( 1.68 - 3.16 ) | 2.27 ( 28.66 ) | 2.15 ( 1.65 ) | 1.1 ( 0.65 ) |
| Pyrexia* | 42 | 5.09 ( 3.63 - 7.16 ) | 4.99 ( 108.95 ) | 4.22 ( 3.18 ) | 2.08 ( 1.59 ) |
| Gait disturbance | 37 | 1.27 ( 0.91 - 1.78 ) | 1.27 ( 2 ) | 1.25 ( 0.95 ) | 0.32 ( -0.16 ) |
| Dizziness* | 34 | 2.28 ( 1.59 - 3.25 ) | 2.25 ( 21.56 ) | 2.13 ( 1.58 ) | 1.09 ( 0.58 ) |
| Influenza like illness* | 32 | 6.94 ( 4.65 - 10.38 ) | 6.83 ( 120.55 ) | 5.4 ( 3.86 ) | 2.43 ( 1.87 ) |
| Nausea* | 32 | 2.11 ( 1.46 - 3.04 ) | 2.09 ( 16.62 ) | 1.99 ( 1.46 ) | 0.99 ( 0.46 ) |
| Asthenia | 31 | 1.38 ( 0.96 - 2 ) | 1.38 ( 3.05 ) | 1.35 ( 1 ) | 0.44 ( -0.09 ) |
| Fall | 29 | 0.6 ( 0.41 - 0.87 ) | 0.6 ( 7.5 ) | 0.62 ( 0.45 ) | -0.7 ( -1.24 ) |
| Multiple sclerosis relapse | 29 | 0.97 ( 0.67 - 1.41 ) | 0.97 ( 0.02 ) | 0.97 ( 0.71 ) | -0.04 ( -0.58 ) |
| Balance disorder | 25 | 1.32 ( 0.88 - 1.98 ) | 1.31 ( 1.79 ) | 1.3 ( 0.92 ) | 0.37 ( -0.21 ) |
| Malaise* | 23 | 2.41 ( 1.56 - 3.73 ) | 2.39 ( 16.87 ) | 2.25 ( 1.57 ) | 1.17 ( 0.55 ) |
| Pain in extremity* | 23 | 1.67 ( 1.09 - 2.56 ) | 1.66 ( 5.64 ) | 1.61 ( 1.13 ) | 0.69 ( 0.07 ) |
| Feeling abnormal* | 23 | 2.64 ( 1.71 - 4.08 ) | 2.62 ( 20.52 ) | 2.44 ( 1.69 ) | 1.28 ( 0.66 ) |
| Covid-19 | 22 | 0.52 ( 0.34 - 0.79 ) | 0.52 ( 9.66 ) | 0.53 ( 0.37 ) | -0.91 ( -1.52 ) |
| Tremor* | 20 | 2.37 ( 1.49 - 3.77 ) | 2.35 ( 14.04 ) | 2.21 ( 1.5 ) | 1.15 ( 0.48 ) |
| Muscular weakness | 20 | 1.54 ( 0.98 - 2.43 ) | 1.53 ( 3.48 ) | 1.5 ( 1.02 ) | 0.58 ( -0.07 ) |
| Urinary tract infection | 20 | 0.64 ( 0.41 - 1.01 ) | 0.65 ( 3.81 ) | 0.66 ( 0.45 ) | -0.6 ( -1.25 ) |
| Inappropriate schedule of product administration* | 20 | 6.22 ( 3.77 - 10.26 ) | 6.16 ( 66.89 ) | 4.98 ( 3.28 ) | 2.32 ( 1.61 ) |
| Myalgia* | 15 | 6.73 ( 3.76 - 12.06 ) | 6.68 ( 55 ) | 5.31 ( 3.26 ) | 2.41 ( 1.6 ) |
| Muscle spasms | 15 | 1.68 ( 0.99 - 2.84 ) | 1.67 ( 3.75 ) | 1.62 ( 1.04 ) | 0.7 ( -0.06 ) |
| Hypoaesthesia | 15 | 1.22 ( 0.72 - 2.06 ) | 1.22 ( 0.55 ) | 1.2 ( 0.78 ) | 0.27 ( -0.48 ) |
| Insomnia* | 14 | 2.39 ( 1.37 - 4.17 ) | 2.38 ( 10.12 ) | 2.24 ( 1.41 ) | 1.16 ( 0.38 ) |
| Diarrhoea | 13 | 0.75 ( 0.43 - 1.3 ) | 0.75 ( 1.06 ) | 0.76 ( 0.48 ) | -0.4 ( -1.19 ) |
| Back pain | 13 | 1.38 ( 0.79 - 2.43 ) | 1.38 ( 1.29 ) | 1.36 ( 0.85 ) | 0.44 ( -0.36 ) |
| Cough | 13 | 1.61 ( 0.92 - 2.84 ) | 1.61 ( 2.8 ) | 1.57 ( 0.98 ) | 0.65 ( -0.16 ) |
| Drug ineffective | 13 | 0.96 ( 0.55 - 1.67 ) | 0.96 ( 0.02 ) | 0.96 ( 0.6 ) | -0.06 ( -0.85 ) |
| Dyspnoea | 12 | 1.17 ( 0.65 - 2.1 ) | 1.17 ( 0.28 ) | 1.16 ( 0.71 ) | 0.21 ( -0.61 ) |
| Alopecia | 12 | 1.51 ( 0.84 - 2.71 ) | 1.5 ( 1.9 ) | 1.47 ( 0.9 ) | 0.56 ( -0.28 ) |
| Nasopharyngitis* | 12 | 2.29 ( 1.26 - 4.17 ) | 2.28 ( 7.83 ) | 2.16 ( 1.31 ) | 1.11 ( 0.26 ) |
| Oropharyngeal pain* | 11 | 4.63 ( 2.4 - 8.9 ) | 4.6 ( 25.48 ) | 3.95 ( 2.29 ) | 1.98 ( 1.07 ) |
| Paraesthesia | 11 | 1.25 ( 0.68 - 2.31 ) | 1.25 ( 0.53 ) | 1.24 ( 0.74 ) | 0.31 ( -0.55 ) |
| Accidental exposure to product* | 11 | 23.16 ( 9.82 - 54.62 ) | 23.02 ( 110.4 ) | 11.49 ( 5.6 ) | 3.52 ( 2.48 ) |
| Pneumonia | 10 | 0.58 ( 0.31 - 1.1 ) | 0.59 ( 2.87 ) | 0.6 ( 0.35 ) | -0.74 ( -1.63 ) |
| Mobility decreased | 9 | 0.8 ( 0.41 - 1.57 ) | 0.8 ( 0.41 ) | 0.81 ( 0.46 ) | -0.3 ( -1.23 ) |
| Rhinorrhoea* | 9 | 4.98 ( 2.4 - 10.31 ) | 4.96 ( 23.01 ) | 4.2 ( 2.28 ) | 2.07 ( 1.06 ) |
| Somnolence* | 9 | 2.1 ( 1.06 - 4.17 ) | 2.09 ( 4.68 ) | 1.99 ( 1.12 ) | 1 ( 0.04 ) |
| Illness | 9 | 1.23 ( 0.63 - 2.42 ) | 1.23 ( 0.37 ) | 1.22 ( 0.69 ) | 0.28 ( -0.66 ) |
| Hypertension | 9 | 1.05 ( 0.53 - 2.05 ) | 1.05 ( 0.02 ) | 1.04 ( 0.6 ) | 0.06 ( -0.88 ) |
| Multiple sclerosis | 9 | 0.35 ( 0.18 - 0.68 ) | 0.35 ( 10.67 ) | 0.36 ( 0.21 ) | -1.46 ( -2.38 ) |
| Injection site pain | 8 | 1.58 ( 0.77 - 3.25 ) | 1.58 ( 1.59 ) | 1.54 ( 0.84 ) | 0.62 ( -0.38 ) |
| Arthralgia | 8 | 0.89 ( 0.44 - 1.8 ) | 0.89 ( 0.11 ) | 0.89 ( 0.49 ) | -0.17 ( -1.15 ) |
| Limb discomfort* | 8 | 2.95 ( 1.4 - 6.19 ) | 2.94 ( 8.98 ) | 2.7 ( 1.45 ) | 1.43 ( 0.41 ) |
| Feeling cold* | 8 | 6.46 ( 2.92 - 14.3 ) | 6.44 ( 28.13 ) | 5.16 ( 2.66 ) | 2.37 ( 1.29 ) |
| Drug effect less than expected* | 8 | 33.64 ( 10.99 - 102.93 ) | 33.49 ( 97 ) | 13.49 ( 5.29 ) | 3.75 ( 2.52 ) |
| Vision blurred | 7 | 1.93 ( 0.89 - 4.2 ) | 1.93 ( 2.87 ) | 1.85 ( 0.97 ) | 0.89 ( -0.18 ) |
| Memory impairment | 7 | 0.44 ( 0.21 - 0.93 ) | 0.44 ( 4.93 ) | 0.45 ( 0.24 ) | -1.15 ( -2.18 ) |
| Pollakiuria* | 7 | 4.59 ( 2.02 - 10.42 ) | 4.58 ( 16.08 ) | 3.94 ( 1.98 ) | 1.98 ( 0.86 ) |

Abbreviation: Asterisks (*) indicate statistically significant signals in algorithm; ROR, reporting odds ratio; PRR, proportional reporting ratio; EBGM, empirical Bayesian geometric mean; EBGM05, the lower limit of the 95% CI of EBGM; IC, information component; IC025, the lower limit of the 95% CI of the IC; CI, confidence interval; PT,preferred term; AEs, adverse events.

Supplementary Table S10: Persistent adverse events for Ofatumumab monotherapy at the PT level from FAERS data.

| PT | Case numbers | ROR(95%Cl) | PRR(χ^2^) | EBGM  (EBGM05) | IC(IC025) |
| --- | --- | --- | --- | --- | --- |
| Fatigue* | 2,658 | 2.08 ( 1.99 - 2.17 ) | 2.02 ( 1199.98 ) | 1.87 ( 1.8 ) | 0.9 ( 0.84 ) |
| Headache* | 2,253 | 2.75 ( 2.63 - 2.88 ) | 2.67 ( 1943.81 ) | 2.35 ( 2.26 ) | 1.23 ( 1.17 ) |
| Pain* | 1,704 | 3 ( 2.85 - 3.17 ) | 2.94 ( 1747.49 ) | 2.53 ( 2.42 ) | 1.34 ( 1.26 ) |
| Chills* | 1,682 | 9.8 ( 9.17 - 10.46 ) | 9.5 ( 6974.07 ) | 5.61 ( 5.31 ) | 2.49 ( 2.4 ) |
| Pyrexia* | 1,650 | 5.26 ( 4.95 - 5.57 ) | 5.11 ( 3786.59 ) | 3.83 ( 3.64 ) | 1.94 ( 1.85 ) |
| Influenza like illness* | 1,416 | 6.52 ( 6.1 - 6.96 ) | 6.36 ( 4113.49 ) | 4.42 ( 4.19 ) | 2.15 ( 2.06 ) |
| Nausea* | 855 | 1.59 ( 1.48 - 1.71 ) | 1.58 ( 162.11 ) | 1.51 ( 1.42 ) | 0.59 ( 0.49 ) |
| Multiple sclerosis relapse | 769 | 0.58 ( 0.53 - 0.62 ) | 0.58 ( 225.59 ) | 0.6 ( 0.57 ) | -0.73 ( -0.84 ) |
| Covid-19 | 731 | 0.59 ( 0.54 - 0.63 ) | 0.59 ( 201.2 ) | 0.61 ( 0.57 ) | -0.71 ( -0.82 ) |
| Asthenia* | 659 | 1.38 ( 1.28 - 1.5 ) | 1.38 ( 61.66 ) | 1.34 ( 1.25 ) | 0.42 ( 0.3 ) |
| Hypoaesthesia* | 645 | 1.3 ( 1.2 - 1.41 ) | 1.3 ( 39.73 ) | 1.27 ( 1.18 ) | 0.34 ( 0.22 ) |
| Gait disturbance | 596 | 1 ( 0.92 - 1.09 ) | 1 ( 0 ) | 1 ( 0.93 ) | 0 ( -0.13 ) |
| Dizziness* | 580 | 1.2 ( 1.1 - 1.3 ) | 1.19 ( 16.56 ) | 1.17 ( 1.09 ) | 0.23 ( 0.11 ) |
| Feeling abnormal* | 579 | 2.04 ( 1.87 - 2.23 ) | 2.03 ( 257.38 ) | 1.87 ( 1.74 ) | 0.9 ( 0.77 ) |
| Malaise* | 575 | 1.84 ( 1.68 - 2.01 ) | 1.83 ( 187.68 ) | 1.71 ( 1.59 ) | 0.78 ( 0.65 ) |
| Pain in extremity* | 556 | 1.47 ( 1.35 - 1.61 ) | 1.47 ( 73.52 ) | 1.41 ( 1.31 ) | 0.5 ( 0.37 ) |
| Nasopharyngitis* | 512 | 1.88 ( 1.71 - 2.07 ) | 1.87 ( 179.87 ) | 1.75 ( 1.62 ) | 0.81 ( 0.67 ) |
| Drug ineffective | 499 | 0.78 ( 0.72 - 0.86 ) | 0.79 ( 27.21 ) | 0.8 ( 0.74 ) | -0.32 ( -0.45 ) |
| Injection site pain* | 490 | 3.55 ( 3.2 - 3.93 ) | 3.52 ( 676.17 ) | 2.92 ( 2.68 ) | 1.55 ( 1.4 ) |
| Inappropriate schedule of product administration* | 467 | 3.59 ( 3.23 - 3.99 ) | 3.57 ( 656.94 ) | 2.95 ( 2.7 ) | 1.56 ( 1.41 ) |
| Arthralgia* | 434 | 1.75 ( 1.58 - 1.94 ) | 1.74 ( 119.54 ) | 1.64 ( 1.51 ) | 0.72 ( 0.57 ) |
| Multiple sclerosis | 425 | 0.45 ( 0.4 - 0.49 ) | 0.45 ( 279.57 ) | 0.47 ( 0.43 ) | -1.09 ( -1.23 ) |
| Back pain* | 424 | 1.6 ( 1.44 - 1.77 ) | 1.59 ( 82.37 ) | 1.52 ( 1.39 ) | 0.6 ( 0.45 ) |
| Myalgia* | 410 | 4.19 ( 3.74 - 4.69 ) | 4.16 ( 721.06 ) | 3.31 ( 3.01 ) | 1.73 ( 1.57 ) |
| Fall | 409 | 0.57 ( 0.52 - 0.63 ) | 0.58 ( 123.83 ) | 0.6 ( 0.55 ) | -0.75 ( -0.89 ) |
| Cough* | 406 | 1.72 ( 1.55 - 1.91 ) | 1.71 ( 104.6 ) | 1.62 ( 1.48 ) | 0.69 ( 0.54 ) |
| Muscular weakness* | 385 | 1.22 ( 1.1 - 1.35 ) | 1.22 ( 13.59 ) | 1.2 ( 1.1 ) | 0.26 ( 0.1 ) |
| Vomiting* | 384 | 1.38 ( 1.24 - 1.54 ) | 1.38 ( 35.6 ) | 1.34 ( 1.22 ) | 0.42 ( 0.26 ) |
| Urinary tract infection | 366 | 0.76 ( 0.68 - 0.84 ) | 0.76 ( 26.23 ) | 0.78 ( 0.71 ) | -0.37 ( -0.52 ) |
| Muscle spasms* | 353 | 1.42 ( 1.27 - 1.58 ) | 1.41 ( 38.26 ) | 1.37 ( 1.25 ) | 0.45 ( 0.29 ) |
| Paraesthesia | 352 | 1.02 ( 0.92 - 1.14 ) | 1.02 ( 0.18 ) | 1.02 ( 0.93 ) | 0.03 ( -0.13 ) |
| Tremor* | 351 | 1.69 ( 1.51 - 1.89 ) | 1.69 ( 85.51 ) | 1.6 ( 1.45 ) | 0.67 ( 0.51 ) |
| Balance disorder | 345 | 0.94 ( 0.84 - 1.05 ) | 0.94 ( 1.26 ) | 0.94 ( 0.86 ) | -0.08 ( -0.24 ) |
| Product dose omission issue | 302 | 0.51 ( 0.45 - 0.57 ) | 0.51 ( 135.04 ) | 0.53 ( 0.48 ) | -0.9 ( -1.07 ) |
| Memory impairment | 298 | 0.63 ( 0.56 - 0.71 ) | 0.64 ( 59.16 ) | 0.66 ( 0.59 ) | -0.61 ( -0.78 ) |
| Oropharyngeal pain* | 298 | 2.54 ( 2.24 - 2.88 ) | 2.53 ( 225.03 ) | 2.25 ( 2.02 ) | 1.17 ( 0.99 ) |
| Accidental exposure* to product | 287 | 22.53 ( 18.44 - 27.53 ) | 22.41 ( 1962.46 ) | 8.15 ( 6.89 ) | 3.03 ( 2.81 ) |
| Injection site bruising* | 285 | 5.52 ( 4.79 - 6.36 ) | 5.5 ( 705.03 ) | 4.02 ( 3.57 ) | 2.01 ( 1.81 ) |
| Illness* | 277 | 1.32 ( 1.16 - 1.49 ) | 1.32 ( 18.81 ) | 1.28 ( 1.15 ) | 0.36 ( 0.18 ) |
| Insomnia* | 272 | 1.21 ( 1.06 - 1.37 ) | 1.2 ( 8.6 ) | 1.19 ( 1.07 ) | 0.24 ( 0.06 ) |
| Migraine* | 257 | 1.73 ( 1.51 - 1.97 ) | 1.72 ( 67.66 ) | 1.63 ( 1.46 ) | 0.7 ( 0.51 ) |
| Diarrhoea | 229 | 0.52 ( 0.46 - 0.6 ) | 0.53 ( 94.33 ) | 0.55 ( 0.49 ) | -0.87 ( -1.07 ) |
| Rhinorrhoea* | 229 | 2.96 ( 2.56 - 3.43 ) | 2.96 ( 235.09 ) | 2.55 ( 2.26 ) | 1.35 ( 1.14 ) |
| Lower respiratory tract infection* | 222 | 8.47 ( 7.11 - 10.08 ) | 8.43 ( 831.68 ) | 5.25 ( 4.53 ) | 2.39 ( 2.16 ) |
| Pruritus | 219 | 0.72 ( 0.63 - 0.83 ) | 0.72 ( 22.15 ) | 0.74 ( 0.66 ) | -0.44 ( -0.64 ) |
| Rash | 218 | 1.01 ( 0.88 - 1.17 ) | 1.01 ( 0.04 ) | 1.01 ( 0.9 ) | 0.02 ( -0.18 ) |
| Limb discomfort* | 212 | 1.8 ( 1.56 - 2.08 ) | 1.8 ( 64.83 ) | 1.69 ( 1.49 ) | 0.75 ( 0.54 ) |
| Stress | 210 | 1.09 ( 0.95 - 1.26 ) | 1.09 ( 1.4 ) | 1.08 ( 0.96 ) | 0.11 ( -0.1 ) |
| Dyspnoea | 210 | 0.75 ( 0.65 - 0.86 ) | 0.75 ( 16.98 ) | 0.76 ( 0.68 ) | -0.39 ( -0.6 ) |
| Somnolence* | 203 | 1.38 ( 1.2 - 1.6 ) | 1.38 ( 19.1 ) | 1.34 ( 1.19 ) | 0.42 ( 0.21 ) |

Abbreviation: Asterisks (*) indicate statistically significant signals in algorithm; ROR, reporting odds ratio; PRR, proportional reporting ratio; EBGM, empirical Bayesian geometric mean; EBGM05, the lower limit of the 95% CI of EBGM; IC, information component; IC025, the lower limit of the 95% CI of the IC; CI, confidence interval; PT,preferred term; AEs, adverse events.
